# KATP Channel Expression Determines ONC212 Sensitivity via Mitochondrial Dysfunction and PERK/ATF4/CHOP Activation in Glioblastoma

**DOI:** 10.1111/jcmm.71301

**Published:** 2026-07-25

**Authors:** Ahmet Taskesen, Ceyhan Hacioglu

**Affiliations:** ^1^ Department of Neurosurgery, Faculty of Medicine Düzce University Düzce Turkey; ^2^ Department of Medical Biochemistry, Faculty of Medicine Düzce University Düzce Turkey

**Keywords:** glioblastoma, integrated stress response, KATP channels, mipridone, mitochondrial dysfunction, ONC212

## Abstract

Glioblastoma (GBM) exhibits profound metabolic plasticity and resistance to conventional therapies, partly driven by mitochondrial adaptability and stress response mechanisms. ONC212, a second‐generation imipridone, targets mitochondrial proteostasis, yet determinants of tumour sensitivity remain unclear. This study aimed to investigate whether ATP‐sensitive potassium (KATP) channel expression modulates ONC212‐induced mitochondrial dysfunction and integrated stress response (ISR) activation in GBM. Human GBM lines (U87, U251, T98G) and non‐malignant SVG p12 astrocytes received ONC212 (0.5–80 μM) for 12–48 h. Viability was assessed by CCK‐8. KATP subunit expression (Kir6.2, SUR1, CCDC51) was quantified by qRT‐PCR and western blot. Mitochondrial ROS quantification, oxygen consumption rate (Seahorse XF), PERK/ATF4/CHOP activation (western blot, immunofluorescence) and apoptosis (caspase‐3/7) were evaluated. KATP was modulated pharmacologically (glibenclamide, diazoxide) and via KCNJ11 (Kir6.2) siRNA. ONC212 induced time‐dependent and tumour‐selective cytotoxicity, with highest sensitivity observed in KATP‐high U87 cells. Treatment significantly increased mitochondrial ROS, impaired oxidative phosphorylation and reduced ATP/ADP ratios, indicating bioenergetic collapse. Concurrently, ONC212 robustly activated the PERK/eIF2α/ATF4/CHOP axis and promoted ATF4 nuclear translocation. PERK inhibition attenuated both stress signalling and cytotoxicity, confirming ISR dependency. KATP inhibition enhanced ONC212‐induced mitochondrial dysfunction, ISR activation and apoptosis, whereas KATP activation exerted protective effects. Importantly, KCNJ11 silencing markedly potentiated ONC212 sensitivity, amplifying ROS production, mitochondrial impairment and caspase‐dependent apoptosis. KATP channel expression may regulate ONC212 responsiveness in GBM by modulating mitochondrial stress and ISR signalling. Targeting KATP channels may enhance imipridone efficacy and represents a promising strategy for metabolically guided GBM therapy.

## 1 Introduction

Glioblastoma (GBM) is recognized as the most aggressive and treatment‐resistant primary tumour of the central nervous system [1]. According to the 2021 World Health Organization (WHO) Classification of Tumours of the Central Nervous System, GBM is defined as a grade 4 diffuse glioma and is classified primarily based on isocitrate dehydrogenase (IDH) status, with IDH‐wildtype GBM representing the vast majority of cases and accounting for the prototypical aggressive clinical course [2]. IDH‐mutant GBM, by contrast, is associated with a more favourable prognosis and is now classified separately from IDH‐wildtype GBM. The revised classification also incorporates molecular markers such as EGFR amplification, TERT promoter mutation and combined whole chromosome 7 gain and chromosome 10 loss (+7/−10) as criteria for the diagnosis of IDH‐wildtype GBM [2]. This molecular stratification has profound implications for patient prognosis and treatment response, highlighting the importance of genetic characterization in GBM research and clinical practice. Standard‐of‐care therapy, which includes extensive surgical resection followed by radiotherapy in combination with temozolomide, provides only limited clinical benefit, with overall survival typically remaining under 15 months [3]. This poor prognosis is largely attributed to the highly heterogeneous nature of GBM, along with its remarkable metabolic adaptability and the rapid emergence of therapeutic resistance mechanisms. Beyond its genomic instability, GBM exhibits extensive metabolic reprogramming that enables tumour cells to dynamically shift between glycolysis and oxidative phosphorylation (OXPHOS), thereby sustaining proliferation under fluctuating microenvironmental conditions [4, 5]. Increasing evidence suggests that subsets of GBM are highly dependent on mitochondrial respiration for survival, stemness maintenance and therapy resistance [6]. This mitochondrial reliance represents a metabolic vulnerability that is not directly targeted by current standard therapies, highlighting the need for strategies that selectively disrupt mitochondrial homeostasis.

ATP‐sensitive potassium (KATP) channels have recently gained prominence as key modulators of cellular energy homeostasis, functioning as metabolic sensors that link intracellular ATP availability to changes in membrane potential and mitochondrial activity [7]. Structurally composed of Kir6.x pore‐forming subunits and regulatory SUR subunits, KATP channels modulate cellular adaptation to energetic stress. In glioma biology, potassium channel expression profiles correlate with tumour proliferation, invasiveness and patient prognosis [8, 9]. Recent findings further suggest that mitochondria‐associated potassium channel components participate in the regulation of mitochondrial membrane potential and reactive oxygen species (ROS) signalling, thereby influencing resistance to metabolic stress [10]. Given the heterogeneous expression of KATP subunits across GBM subtypes, it is plausible that tumours enriched in KATP infrastructure exhibit a distinct mitochondrial phenotype that may determine sensitivity to targeted metabolic disruption.

Endoplasmic reticulum (ER) stress and mitochondrial dysfunction are central components of the integrated stress response (ISR), a conserved adaptive program activated in response to proteotoxic, oxidative and metabolic insults [11]. In GBM, chronic hypoxia, nutrient limitation and rapid proliferative demand create a microenvironment that persistently engages ER stress signalling pathways [12, 13]. Engagement of ER stress signalling pathways, particularly through activation of protein kinase RNA‐like ER kinase (PERK), leads to phosphorylation of eIF2α, suppression of overall protein synthesis and preferential upregulation of stress‐responsive transcription factors such as ATF4 and CHOP. These effectors regulate genes involved in redox balance, amino acid metabolism and apoptosis, thereby determining whether tumour cells undergo adaptive survival or programmed cell death [14]. Notably, PERK signalling has been shown to promote GBM cell survival under hypoxic and metabolic stress conditions, contributing to therapy resistance and tumour progression [12]. Sustained ER stress in GBM also disrupts mitochondrial homeostasis through Ca^2+^ flux alterations, increased ROS production and loss of mitochondrial membrane potential, ultimately engaging intrinsic apoptotic pathways [11]. This ER–mitochondria crosstalk is mediated in part through mitochondria‐associated membranes (MAMs), which coordinate calcium signalling and metabolic adaptation in glioma cells. Collectively, these findings indicate that the PERK/ATF4/CHOP axis constitutes a critical stress‐adaptation hub in GBM and represents a therapeutically exploitable vulnerability linking ER stress to mitochondrial dysfunction.

The selection of GBM cell lines for this study was based on their well‐characterized genetic heterogeneity and divergent metabolic phenotypes. U87MG, despite harbouring mutations in PTEN and CDKN2A, exhibits wild‐type TP53 and IDH1 and displays a classic proneural‐like gene expression profile with relatively high mitochondrial respiratory capacity [15]. In contrast, U251 carries mutant TP53 (p.R273H) and wild‐type IDH1, with a mesenchymal‐like transcriptional signature associated with enhanced glycolytic metabolism and increased therapy resistance [16]. T98G is characterized by mutant TP53 (p.P151S), wild‐type IDH1 and MGMT promoter unmethylated status, rendering it relatively resistant to alkylating agents and exhibiting pronounced metabolic plasticity [17]. These genetic distinctions, particularly the TP53 status, may influence cellular stress responses and metabolic flexibility, as p53 is known to regulate mitochondrial respiration, antioxidant defence and the integrated stress response [18]. Importantly, MGMT promoter methylation status, which is associated with improved temozolomide responsiveness, varies across these cell lines, with U87 and U251 reported to exhibit MGMT promoter methylation, while T98G is characteristically unmethylated [17]. This genetic heterogeneity provides an ideal platform to evaluate the determinants of ONC212 sensitivity across distinct GBM molecular subtypes.

Imipridone derivative ONC212 has emerged as a next‐generation anti‐cancer agent with enhanced potency compared to first‐generation imipridones. Unlike ONC201, which primarily targets dopamine receptor D2 (DRD2), ONC212 has been shown to exert its anti‐tumour activity predominantly through activation of the mitochondrial caseinolytic protease P (ClpP) and engagement of G protein–coupled receptor 132 (GPR132), a stress‐responsive receptor implicated in tumour cell survival [19, 20]. ONC212 allosterically activates ClpP within the mitochondrial matrix, leading to accelerated degradation of respiratory chain components and other essential mitochondrial proteins. This proteolytic hyperactivation results in impaired OXPHOS, mitochondrial membrane depolarization, excessive ROS production and bioenergetic collapse [21].

ONC212‐driven disruption of mitochondrial function initiates the integrated stress response (ISR), marked by eIF2α phosphorylation and the subsequent activation of ATF4‐ and CHOP‐dependent transcriptional networks [19]. Sustained ISR activation promotes apoptotic signalling when mitochondrial proteostasis cannot be restored. Preclinical studies have demonstrated that ONC212 exhibits broad anti‐tumour efficacy across multiple malignancies, including hematologic cancers and solid tumours, with significantly greater potency than ONC201 in OXPHOS‐dependent cellular contexts [21]. These findings position ONC212 as a potent mitochondrial stress inducer capable of selectively targeting metabolically vulnerable tumour subpopulations. Although ONC212 has shown robust preclinical efficacy, the determinants of tumour susceptibility remain incompletely defined. Given that ATP‐sensitive potassium (KATP) channels function as metabolic sensors that couple intracellular ATP levels to membrane excitability and mitochondrial homeostasis, it is plausible that KATP expression profiles influence cellular adaptation to ONC212‐induced mitochondrial proteotoxic stress. Tumours exhibiting elevated mitochondrial respiration and enhanced reliance on OXPHOS may be particularly susceptible to ClpP hyperactivation and ISR engagement.

In this study, we aimed to determine whether ONC212 treatment induces mitochondrial dysfunction and ISR activation in GBM cells in a manner that correlates with KATP channel expression. We hypothesized that GBM cell populations with elevated KATP channel activity and increased metabolic reliance on mitochondrial OXPHOS are predisposed to ONC212‐mediated mitochondrial proteolytic stress, bioenergetic collapse and activation of PERK/ATF4/CHOP signalling. To test this hypothesis, we employed a series of functional assays including cellular viability profiling, mitochondrial respiration analysis, evaluation of ER stress markers and KATP expression quantification to define the mechanistic basis of ONC212 sensitivity in GBM.

## 2 Materials and Methods

### 2.1 Reagents and Chemicals

The imipridone compound ONC212 (catalogue ab287143) was sourced from Abcam (Cambridge, UK). Glibenclamide (G0639), diazoxide (D9035) and dimethyl sulfoxide (DMSO, D2650) were acquired from Sigma‐Aldrich (St. Louis, MO, USA). ISRIB (SML0843) and the PERK inhibitor GSK2606414 (SML2481) were also obtained from Sigma‐Aldrich. All substances were solubilized in DMSO to generate stock solutions as follows: ONC212 at 20 mM, glibenclamide at 50 mM, diazoxide at 100 mM, ISRIB at 10 mM and GSK2606414 at 10 mM. These stocks were kept at −20°C in darkness. Prior to each experiment, working concentrations were prepared by diluting the stocks in culture medium immediately before use. The final DMSO concentration in every treatment (including vehicle controls) never exceeded 0.1% (v/v).

### 2.2 Cell Culture

The human GBM lines U87, U251 and T98G were procured from the American Type Culture Collection (ATCC, USA). Non‐malignant control cells consisted of SVG p12 human foetal glial cells (ATCC CRL‐8621). All cell lines were maintained in Dulbecco's Modified Eagle's Medium containing high glucose (DMEM, Gibco, 11,965,092, Waltham, MA, USA) supplemented with 10% foetal bovine serum (FBS, Gibco, 10,270,106), 100 U/mL penicillin, and 100 μg/mL streptomycin (Gibco, 15,140,122). Incubation conditions were 37°C with 5% CO_2_ in a humidified atmosphere. Routine subculturing was performed at 80% confluence using 0.25% trypsin–EDTA (Gibco, 25,200,056). Only mycoplasma‐free cells in the logarithmic growth phase were used for experiments.

### 2.3 CCK‐8 Viability Assay

Cells were plated in 96‐well plates at 5 × 10^3^ cells per well and left to attach overnight. Subsequently, they were exposed to a logarithmic concentration series of ONC212 (0.5, 1, 2.5, 5, 10, 20, 40 and 80 μM) for 12, 24, or 48 h. Control wells received vehicle (DMSO) at a concentration matching that present in the highest drug treatment. Viability was determined with the Cell Counting Kit‐8 (CCK‐8, BMU106‐EN) following the manufacturer's instructions. Briefly, 10 μL of CCK‐8 solution was added to each well containing 100 μL of culture medium, followed by a 2‐h incubation at 37°C. Absorbance at 450 nm was measured with a microplate reader (BioTek Instruments, USA), after subtracting background from cell‐free wells. Since the CCK‐8 assay measures mitochondrial metabolic activity, the results are interpreted as a metabolic sensitivity index rather than absolute cell death. SVG p12 astrocytes were analysed in parallel to evaluate tumour‐selective toxicity. The selectivity index (SI) was calculated as the ratio of the half‐maximal inhibitory concentration (IC_50_) in normal cells to that in cancer cells: SI = IC_50_ (normal)/IC_50_ (cancer).

### 2.4 Cell Transfection

Cells were seeded at 60% confluence and transfected with 50 nM small interfering RNA (siRNA) using Lipofectamine RNAiMAX according to the product manual. After 6 h, the medium was replaced with fresh complete medium. Experiments commenced 24 h post‐transfection. The siRNA duplex targeting KCNJ11 (Kir6.2) had the following sequences: sense 5′‐GCU UCU UCU UCG UGA UGA AUU‐3′, antisense 5′‐UU CAU CAC GAA GAA GAA GCU U‐3′. A scrambled control siRNA (sense 5′‐UUC UCC GAA CGU GUC ACG UUU‐3′, antisense 5′‐ACG UGA CAC GUU CGG AGA AUU‐3′) was used as a negative control.

### 2.5 Caspase‐3/7 Activity Analysis

Caspase‐3/7 activation was measured with the Muse Caspase‐3/7 Assay Kit (MCH100108, USA), which detects effector caspases active during the execution phase of apoptosis. To simultaneously assess plasma membrane integrity, 7‐aminoactinomycin D (7‐AAD) was included as a cell‐impermeant dye, enabling discrimination among early apoptotic, late apoptotic and non‐viable populations. Cells were seeded in 24‐well plates at 1 × 10^5^ cells per well and treated with ONC212 for 24 h. Following treatment, both adherent and detached cells were collected by gentle trypsinization, pooled and washed twice with cold phosphate‐buffered saline (PBS). Cell pellets were resuspended in Muse assay buffer to a density of approximately 1 × 10^5^ cells/mL. Then, 5 μL of the Caspase‐3/7 reagent was added to 100 μL of the cell suspension and incubated for 30 min at 37°C in the dark, allowing intracellular binding to cleaved caspase‐3/7 products. Afterwards, 150 μL of 7‐AAD was added, mixed and samples were immediately analysed on a Muse Cell Analyser.

### 2.6 Quantitative Real‐Time PCR (qRT‐PCR)

Total RNA was extracted from cultured cells using the RNeasy Plus Mini Kit (74,134, Qiagen, Germany) as per the manufacturer's protocol. RNA concentration and purity were assessed on a NanoDrop 2000 spectrophotometer (Thermo Fisher Scientific, Waltham, MA, USA); all samples had A_2_₆₀/A_2_₈₀ ratios between 1.9 and 2.1. Complementary DNA (cDNA) was synthesized from 1 μg of total RNA using the High‐Capacity cDNA Reverse Transcription Kit (4,368,814, Applied Biosystems, Foster City, CA, USA) in a 20 μL reaction volume. The following primer sequences were employed: EIF2AK3 (PERK) forward 5′‐TGG GCA GAA GGA GGA GCA GGA T‐3′, reverse 5′‐AGC CAG GCA GCA GGT CCT TCA‐3′; ATF4 forward 5′‐GCC TCA GAT GCC ATG ACC GAA A‐3′, reverse 5′‐CGA AGT GGA GGC TGG GGA CAT A‐3′; DDIT3 (CHOP) forward 5′‐GGA GCT GGA AGC CTG GTA TGA G‐3′, reverse 5′‐TCC CTG GTC AGG CGC TCG ATT‐3′; KCNJ11 (Kir6.2) forward 5′‐ACG GTG GTC ATC ATC GTG GAG C‐3′, reverse 5′‐GCC ATG GAT GAT GCA CCA CAG G‐3′; ABCC8 (SUR1) forward 5′‐GCA GGC AGA AGG TGT TGG CCA‐3′, reverse 5′‐CGG TAG GTG ATG GTG AGG TTC GG‐3′; CCDC51 forward 5′‐CGG CAG TAG ACG CTG GAC GC‐3′, reverse 5′‐CCG GGA CAT GAC CTC CAG GG‐3′; GAPDH forward 5′‐GCT AAG GTG AAG GTC GGA GTG CC‐3′, reverse 5′‐GCT AAG ATG GTG ATG GGA TGG TC‐3′. qRT‐PCR was carried out with PowerUp SYBR Green Master Mix (A25742, Applied Biosystems) on a QuantStudio 5 Real‐Time PCR System (Applied Biosystems). Each 10 μL reaction contained 5 μL of 2× SYBR Green Master Mix, 1 μL of diluted cDNA (equivalent to 10 ng of input RNA), 0.5 μL each of forward and reverse primers (final concentration 250 nM) and 3 μL of nuclease‐free water. All reactions were run in technical triplicates from seven independent biological replicates. Thermal cycling conditions were: 95°C for 2 min (polymerase activation), then 40 cycles of 95°C for 15 s and 60°C for 1 min (annealing/extension). Relative expression was calculated using the comparative Ct (2^−^ΔΔCt) method.

### 2.7 Western Blotting

Protein lysates were prepared with RIPA Lysis and Extraction Buffer (89,900, Thermo Fisher Scientific) supplemented with Phosphatase Inhibitor Cocktail (78,442, Thermo Fisher Scientific). After treatment, cells were washed twice with ice‐cold PBS and lysed directly in culture dishes using ice‐cold RIPA buffer (100 μL per well of a 6‐well plate). Lysates were scraped, transferred to microcentrifuge tubes and kept on ice for 30 min with intermittent vortexing. Insoluble debris was pelleted by centrifugation at 14,000 × g for 15 min at 4°C, and the supernatants were collected. Protein concentrations were measured with the BCA Protein Assay Kit (23,225, Thermo Fisher Scientific) following the microplate protocol. Bovine serum albumin (BSA) standards were prepared in the same buffer as the samples, and absorbance was read at 562 nm on a microplate reader (BioTek). Equal amounts of protein (20 μg per lane) were separated by SDS‐PAGE using 10% gels. Electrophoresis was run at 120 V for 60 min until the dye front reached the gel bottom. Proteins were transferred onto PVDF membranes. Primary antibodies used were: anti‐phospho‐PERK (Thr980) (3179, Cell Signalling Technology [CST], 1:500), anti‐phospho‐eIF2α (Ser51) (3398, CST, 1:500), anti‐ATF4 (11,815, CST, 1:500), anti‐CHOP (2895, CST, 1:500), anti‐Kir6.2 (PA5‐99440, Invitrogen, 1:2000), anti‐SUR1 (ab32844, Abcam, 1:500), anti‐CCDC51 (PA5‐52767, Thermo Fisher Scientific, 1:500), anti‐cleaved caspase‐3 (Asp175) (9661, CST, 1:500) and anti‐GAPDH (5174, CST, 1:5000) as a loading control. After primary antibody incubation, membranes were washed three times for 10 min and then incubated with HRP‐conjugated secondary antibodies for 1 h at room temperature. Protein bands were visualized using ECL Substrate (1,705,060, Bio‐Rad) as directed.

### 2.8 Mitochondrial ROS Production

Mitochondrial superoxide generation was detected using MitoSOX Red Mitochondrial Superoxide Indicator (M36008, Invitrogen). Cells were seeded in 24‐well plates at 1 × 10^5^ cells per well and treated with ONC212 as described. Following treatment, cells were incubated with 5 μM MitoSOX Red in Hank's Balanced Salt Solution (HBSS, 14025092, Gibco) containing calcium and magnesium for 20 min at 37°C in the dark. Cells were then washed gently three times with warm HBSS, and fluorescence was immediately quantified by fluorescence microscopy. Images were captured with a Zeiss Axio Observer 7 microscope using a 40× objective and identical exposure settings across all conditions. For each independent experiment (*n* = 7), at least 50 cells per condition were randomly selected and analysed in a blinded manner using ImageJ.

### 2.9 Seahorse XF Analysis

Mitochondrial respiratory function was evaluated with a Seahorse XFe96 Extracellular Flux Analyser (Agilent Technologies) using the standard Mito Stress Test protocol. Cells were seeded in Seahorse XFe96 cell culture microplates at an optimized density of 1 × 10^4^ cells per well and allowed to attach overnight to achieve a confluent monolayer and linear oxygen consumption rate (OCR) responses. To assess early bioenergetic changes, cells were treated with ONC212 for 24 h. One hour before the assay, the culture medium was replaced with Seahorse XF DMEM assay medium (Agilent) supplemented with 10 mM glucose, 1 mM pyruvate, and 2 mM L‐glutamine (pH 7.4). Plates were then incubated at 37°C in a non‐CO_2_ incubator for 1 h to allow temperature and pH equilibration. OCR was measured under basal conditions and after sequential injections of oligomycin (1 μM), FCCP (1.5 μM), and rotenone plus antimycin A (0.5 μM each). Each measurement cycle consisted of 3 min of mixing, 2 min of waiting and 3 min of measurement. After the assay, protein content per well was determined by BCA assay to normalize OCR values. Data were analysed with Wave software (version 2.6, Agilent), and the following parameters were calculated: basal respiration, ATP‐linked respiration, maximal respiration, spare respiratory capacity and ATP/ADP ratio, each expressed as pmol/min/μg protein.

### 2.10 ATF4 Nuclear Translocation (Immunofluorescence)

ATF4 nuclear translocation was assessed by immunofluorescence. Cells grown on glass coverslips were treated with ONC212 as described, then fixed with 4% paraformaldehyde for 15 min at room temperature. After three washes with PBS, cells were permeabilized with 0.1% Triton X‐100 (T8787, Sigma‐Aldrich) in PBS for 10 min at room temperature. Blocking was performed with 5% normal goat serum (16,210,072, Gibco) in PBS for 1 h at room temperature, followed by overnight incubation at 4°C with anti‐ATF4 antibody (11,815, Cell Signalling Technology) diluted 1:200 in blocking buffer. Cells were then washed three times with PBS and incubated with Alexa Fluor 488‐conjugated goat anti‐rabbit secondary antibody (A11008, Invitrogen, 1:500) for 1 h at room temperature in the dark. Nuclei were counterstained with DAPI (4′,6‐diamidino‐2‐phenylindole, D1306, Invitrogen, 1 μg/mL) for 5 min. Confocal images were acquired with a Zeiss LSM 880 microscope using a 40× objective. Nuclear ATF4 localization was quantified by measuring mean fluorescence intensity in nuclear regions (defined by DAPI staining) relative to cytoplasmic regions using ImageJ. At least 50 cells per condition from seven independent experiments were analysed in a blinded manner.

### 2.11 Statistical Analysis

Data are presented as mean ± SEM from at least seven independent experiments. Statistical analyses were performed using GraphPad Prism software. Normality was assessed using the Shapiro–Wilk test. Comparisons among multiple groups were conducted using one‐way or two‐way ANOVA followed by Tukey's post hoc test. For two‐group comparisons, an unpaired Student's *t*‐test was applied. IC_50_ values were calculated using nonlinear regression based on a four‐parameter logistic model. Statistical significance was defined as *p* < 0.05.

## 3 Results

### 3.1 ONC212 Induces Time‐Dependent and Tumour‐Selective Cytotoxicity

**FIGURE 1:**
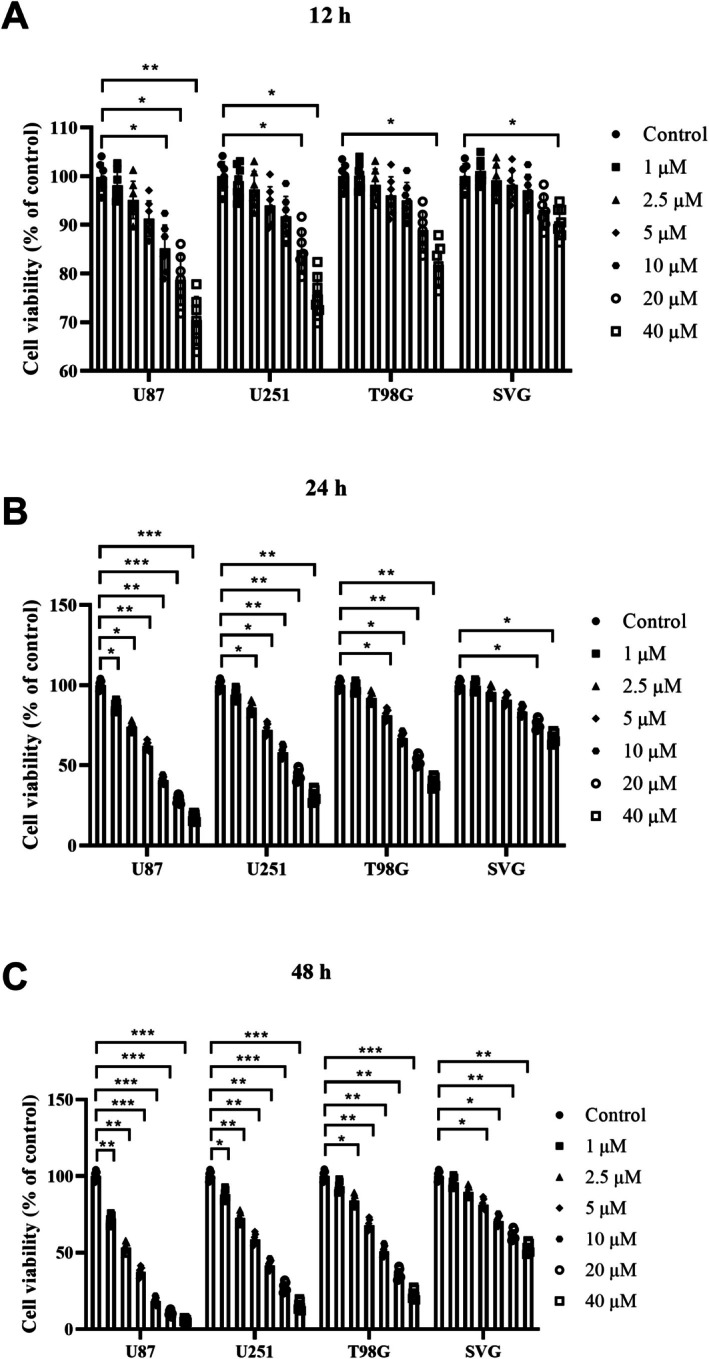
ONC212 induces time‐dependent and tumour‐selective cytotoxicity in GBM cells. U87, U251, and T98G glioblastoma cells and non‐malignant SVG p12 astrocytes were treated with increasing concentrations of ONC212 (1–40 μM) for 12 h, 24 h, and 48 h, and cell viability was assessed using the CCK‐8 assay. (A) At 12 h, ONC212 produced minimal cytotoxic effects across GBM cell lines, indicating early stress signalling without substantial loss of viability. (B) At 24 h, a concentration‐dependent reduction in viability emerged, with U87 cells showing the highest sensitivity compared with U251 and T98G cells, while SVG p12 astrocytes remained relatively resistant. (C) At 48 h, ONC212‐induced cytotoxicity was markedly enhanced, demonstrating a clear time‐dependent effect and increased tumour selectivity, particularly in U87 cells. Data are presented as mean ± SEM from at least seven independent experiments. Statistical significance was determined using two‐way ANOVA. *p < 0.05, **p < 0.001, ***p < 0.0001.

To determine temporal cytotoxic dynamics and tumour selectivity, U87, U251, T98G and non‐malignant SVG p12 astrocytes were treated with increasing concentrations of ONC212 (1, 2.5, 5, 10, 20 and 40 μM) for 12, 24 and 48 h (Figure 1).

At 12 h, ONC212 induced minimal cytotoxicity across all GBM cell lines, indicating early signalling engagement without overt viability loss (Figure 1A). In U87 cells, viability remained near control levels at lower concentrations: 98% ± 2.5% at 1 μM (*p* = 0.512), 95% ± 2.9% at 2.5 μM (*p* = 0.231), and declined modestly to 91% ± 2.8% at 5 μM (*p* = 0.084) and 86% ± 3.2% at 10 μM (*p* = 0.041). At higher concentrations, viability decreased to 79% ± 3.5% at 20 μM (*p* = 0.023) and 71% ± 3.8% at 40 μM (*p* = 0.009). The calculated IC_50_ was 28.4 ± 2.1 μM. U251 cells maintained 99% ± 2.7% viability at 1 μM (*p* = 0.678), 97% ± 3.1% at 2.5 μM (*p* = 0.342), 94% ± 3.3% at 5 μM (*p* = 0.112), 92% ± 3.5% at 10 μM (*p* = 0.097), with further reductions to 85% ± 3.9% at 20 μM (*p* = 0.041) and 76% ± 4.2% at 40 μM (*p* = 0.018), yielding an IC_50_ of 34.6 ± 2.8 μM. T98G cells were even less responsive: viability was 100% ± 2.4% at 1 μM (*p* = 0.894), 98% ± 2.8% at 2.5 μM (*p* = 0.456), 96% ± 3.0% at 5 μM (*p* = 0.201), 95% ± 3.1% at 10 μM (*p* = 0.124), 89% ± 3.4% at 20 μM (*p* = 0.058) and 82% ± 3.7% at 40 μM (*p* = 0.032), with an IC_50_ of 41.2 ± 3.4 μM. Non‐malignant SVG p12 astrocytes exhibited 101% ± 2.3% viability at 1 μM (*p* = 0.923), 99% ± 2.6% at 2.5 μM (*p* = 0.612), 98% ± 2.8% at 5 μM (*p* = 0.378), 97% ± 2.9% at 10 μM (*p* = 0.212), 93% ± 3.1% at 20 μM (*p* = 0.089) and 88% ± 3.3% at 40 μM (*p* = 0.047), with an IC_50_ exceeding 50 μM. The calculated selectivity indices (SI) at 12 h were modest (> 1.76 for U87, > 1.44 for U251 and > 1.21 for T98G), indicating limited tumour selectivity at early exposure (Table S1).

Clear differential sensitivity emerged at 24 h. In U87 cells, viability decreased progressively (Figure 1B): 87% ± 2.6% at 1 μM (*p* = 0.032), 74% ± 2.4% at 2.5 μM (*p* = 0.008), 62% ± 2.3% at 5 μM (*p* = 0.0005), 41% ± 1.9% at 10 μM (*p* = 0.0002), 29% ± 1.8% at 20 μM (*p* = 0.0001) and 18% ± 1.5% at 40 μM (*p* = 0.0001), yielding an IC_50_ of 7.8 ± 0.5 μM. U251 cells exhibited intermediate sensitivity: 94% ± 3.2% at 1 μM (*p* = 0.154), 86% ± 3.4% at 2.5 μM (*p* = 0.048), 72% ± 3.5% at 5 μM (*p* = 0.012), 58% ± 3.6% at 10 μM (*p* = 0.0041), 44% ± 3.8% at 20 μM (*p* = 0.0012) and 31% ± 4.0% at 40 μM (p = 0.0005), with an IC_50_ of 13.6 ± 0.9 μM. T98G cells remained comparatively resistant: 98% ± 3.0% at 1 μM (*p* = 0.432), 92% ± 3.1% at 2.5 μM (*p* = 0.089), 81% ± 3.2% at 5 μM (*p* = 0.031), 67% ± 3.1% at 10 μM (*p* = 0.0184), 53% ± 3.3% at 20 μM (*p* = 0.0052) and 39% ± 3.5% at 40 μM (*p* = 0.0018), with an IC_50_ of 19.2 ± 1.2 μM. In contrast, SVG p12 astrocytes displayed significantly greater resistance: 99% ± 2.8% at 1 μM (*p* = 0.623), 96% ± 2.9% at 2.5 μM (*p* = 0.231), 91% ± 2.8% at 5 μM (p = 0.089), 84% ± 2.7% at 10 μM (*p* = 0.112), 76% ± 2.9% at 20 μM (*p* = 0.043) and 67% ± 3.1% at 40 μM (*p* = 0.021), with an IC_50_ of 26.8 ± 2.3 μM. Selectivity index analysis at 24 h revealed enhanced tumour selectivity, particularly in U87 cells (SI = 3.44), followed by U251 (SI = 1.97) and T98G (SI = 1.40) (Table S1).

At 48 h, cytotoxicity intensified further across GBM models. U87 cells demonstrated pronounced sensitivity (Figure 1C): viability was 72% ± 2.2% at 1 μM (*p* = 0.004), 54% ± 2.0% at 2.5 μM (*p* = 0.0003), 38% ± 1.7% at 5 μM (*p* = 0.0001), 19% ± 1.3% at 10 μM (*p* = 0.0001), 11% ± 1.1% at 20 μM (*p* = 0.0001) and 6% ± 0.8% at 40 μM (*p* = 0.0001), corresponding to an IC_50_ of 4.5 ± 0.4 μM. U251 cells showed 88% ± 2.9% at 1 μM (*p* = 0.041), 73% ± 3.0% at 2.5 μM (*p* = 0.009), 59% ± 2.9% at 5 μM (*p* = 0.0023), 42% ± 2.8% at 10 μM (*p* = 0.0013), 28% ± 2.7% at 20 μM (*p* = 0.0006) and 16% ± 2.5% at 40 μM (*p* = 0.0003), with an IC_50_ of 9.1 ± 0.7 μM. T98G cells retained 93% ± 3.1% at 1 μM (*p* = 0.102), 84% ± 3.2% at 2.5 μM (*p* = 0.027), 68% ± 3.2% at 5 μM (*p* = 0.0098), 51% ± 3.2% at 10 μM (*p* = 0.0062), 36% ± 3.3% at 20 μM (*p* = 0.0021) and 23% ± 3.1% at 40 μM (*p* = 0.0008), with an IC_50_ of 15.2 ± 1.1 μM. Although SVG p12 astrocytes exhibited some reduction in viability at 48 h (96% ± 2.9% at 1 μM [*p* = 0.312], 90% ± 2.9% at 2.5 μM [*p* = 0.087], 82% ± 3.0% at 5 μM [*p* = 0.042], 71% ± 3.0% at 10 μM [p = 0.041], 62% ± 3.1% at 20 μM [*p* = 0.022] and 53% ± 3.2% at 40 μM [*p* = 0.011]), they remained substantially less sensitive than malignant counterparts, with an IC_50_ of 23.4 ± 2.0 μM. Selectivity indices at 48 h further increased, reaching 5.20 for U87, 2.57 for U251 and 1.54 for T98G cells (Table S1).

Collectively, these findings demonstrate that ONC212 exerts a clear time‐dependent and tumour‐selective cytotoxic effect in GBM models. While early exposure primarily reflects stress signalling without major viability loss, prolonged treatment results in progressive cytotoxic execution, particularly in U87 cells. The increasing selectivity index over time indicates an expanding therapeutic window, supporting 24‐h exposure as an optimal mechanistic time point and 48‐h exposure as a robust endpoint for cytotoxic assessment.

### 3.2 Baseline KATP Subunit Expression Reveals Intrinsic Metabolic Heterogeneity Across GBM Cell Lines

**FIGURE 2:**
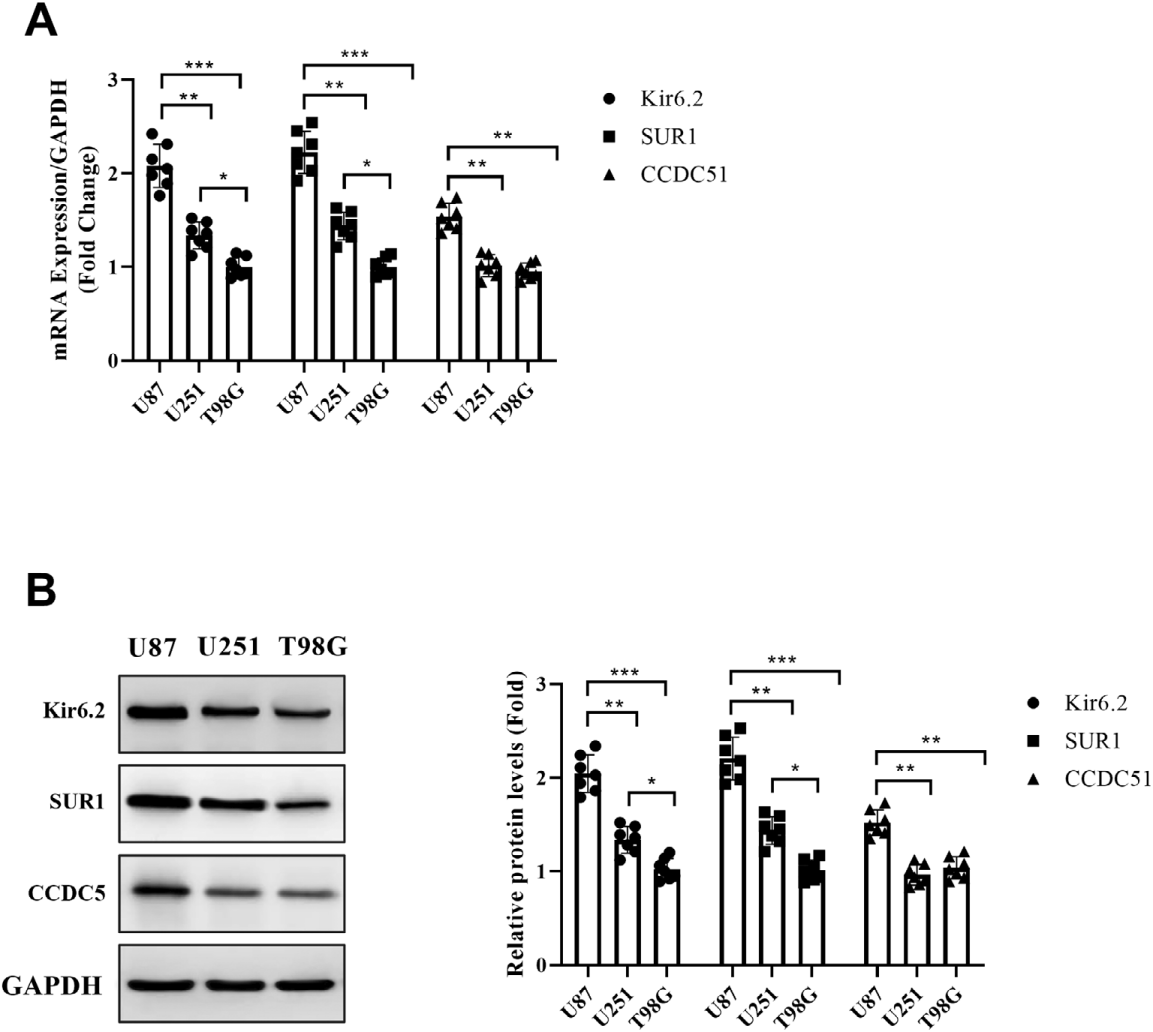
Baseline expression of KATP channel subunits differs among GBM cell lines. Expression levels of KATP channel components were evaluated in U87, U251, and T98G glioblastoma cells. (A) qRT‐PCR analysis of the plasmalemmal KATP channel subunits KCNJ11 (Kir6.2) and ABCC8 (SUR1), as well as the mitochondrial‐associated KATP component CCDC51, demonstrating differential transcript expression across GBM cell lines. Gene expression levels were normalized to GAPDH. (B) Representative Western blot analysis showing protein expression of Kir6.2, SUR1, and CCDC51 in the indicated cell lines, with GAPDH used as a loading control. Corresponding densitometric quantification of protein bands normalized to GAPDH is shown. Data are presented as mean ± SEM from seven independent experiments. Statistical significance was determined using two‐way ANOVA. **p < 0.01, ***p < 0.001.

Baseline expression levels of KATP channel subunits were evaluated in untreated U87, U251 and T98G GBM cell lines. qRT‐PCR analysis demonstrated significantly higher transcript levels of KCNJ11 in U87 cells (2.08 ± 0.14) compared with T98G cells (1.00 ± 0.09; *p* = 0.0004) and U251 cells (1.34 ± 0.11; *p* = 0.0021; Figure 2A). Similarly, ABCC8 expression was markedly elevated in U87 cells (2.21 ± 0.18) relative to T98G (1.00 ± 0.08; *p* = 0.0003) and U251 (1.42 ± 0.13; *p* = 0.0018). CCDC51 transcript levels were also increased in U87 cells (1.52 ± 0.12) compared with U251 (1.00 ± 0.10; *p* = 0.0067).

Protein analysis corroborated these findings. Kir6.2 abundance in U87 cells (2.03 ± 0.16) was significantly higher than in T98G cells (1.00 ± 0.11; *p* = 0.0006; Figure 2B). Likewise, SUR1 expression was elevated in U87 (2.18 ± 0.21) relative to T98G (1.00 ± 0.09; *p* = 0.0004). CCDC51 protein levels were also significantly increased in U87 (1.49 ± 0.13) compared with U251 (1.00 ± 0.12; *p* = 0.0082). Collectively, these results demonstrate a clear KATP expression gradient (U87 > U251 > T98G), indicating intrinsic metabolic heterogeneity among GBM models.

### 3.3 ONC212 Induces Mitochondrial ROS Accumulation and Bioenergetic Collapse

To determine whether ONC212 (5 μM) disrupts mitochondrial redox balance and cellular bioenergetics, mitochondrial superoxide levels and mitochondrial respiration parameters were evaluated following 24 h of treatment.

**FIGURE 3:**
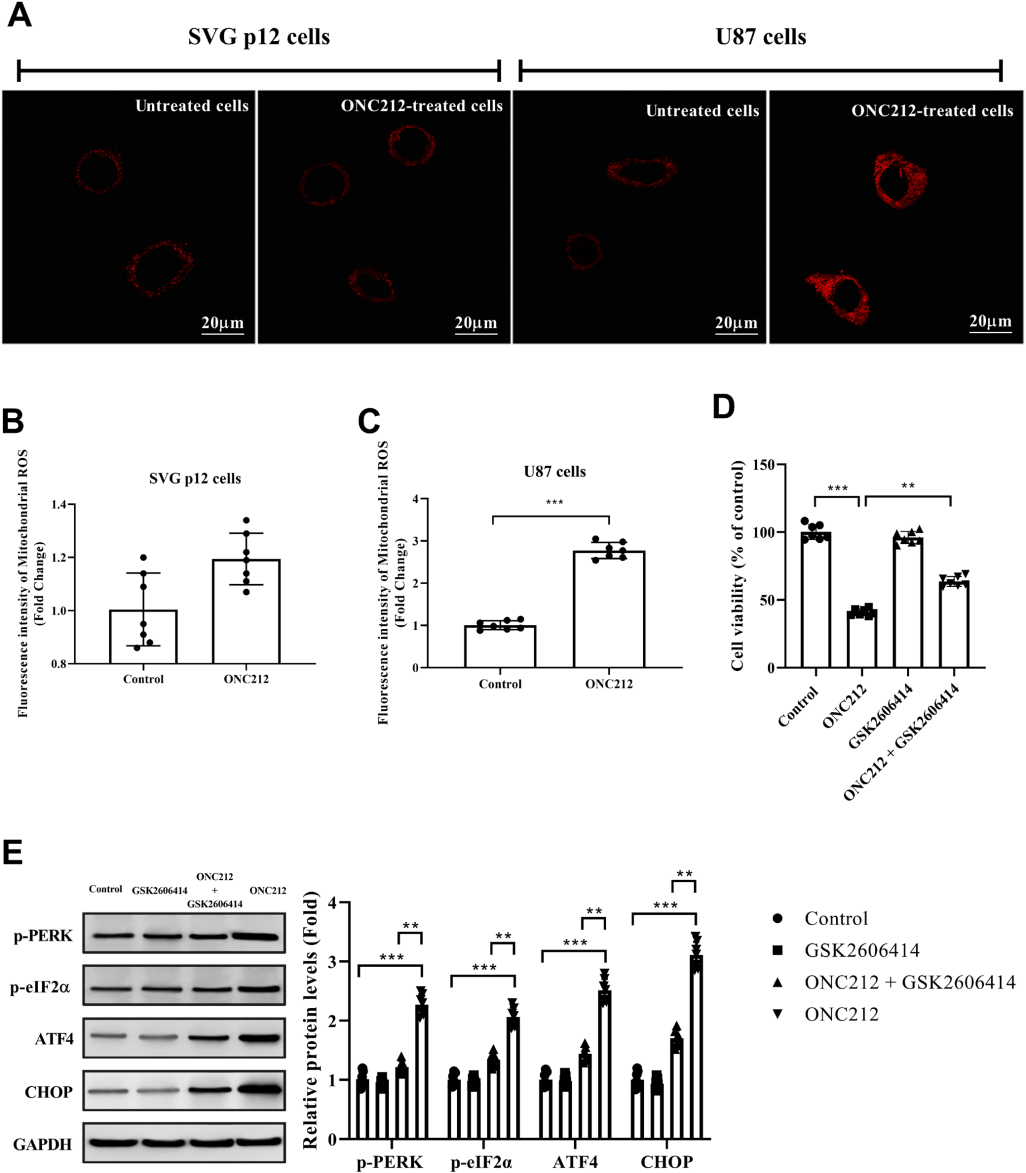
ONC212 induces mitochondrial ROS production in both SVG p12 and U87 cells. ONC212 activates the PERK/eIF2α/ATF4/CHOP axis of the integrated stress response in U87 cells. (A) Representative confocal images of MitoSOX Red staining showing mitochondrial superoxide levels in untreated and ONC212‐treated SVG p12 and U87 cells. Scale bar = 20 μm. (B, C) Quantitative analysis of mitochondrial ROS fluorescence intensity. ONC212 treatment significantly elevates MitoSOX fluorescence compared to untreated controls in both cell lines. (D) Cell viability was assessed by CCK‐8 assay in U87 cells treated with vehicle (Control), ONC212 (10 μM, 6 h), GSK2606414 (a selective PERK inhibitor), or the combination of ONC212 and GSK2606414. (E) Quantitative analysis of relative protein levels (fold change) for p‐PERK, p‐eIF2α, ATF4, and CHOP under the indicated conditions. Data are presented as mean ± SEM from at least seven independent experiments. Statistical significance was assessed using Student's *t*‐tests. ***p < 0.001.

Mitochondrial superoxide production was first assessed using a mitochondrial ROS‐sensitive probe (Figure 3A,C). In U87 cells, basal mitochondrial ROS levels were low in controls (1.00 ± 0.08 fold). Exposure to ONC212 resulted in a significant elevation of mitochondrial superoxide levels, reaching 2.53 ± 0.25 fold relative to control cells (*p* < 0.0001), indicating pronounced oxidative stress within the mitochondrial compartment.

In contrast, SVG p12 astrocytes, representing non‐malignant brain cells, displayed minimal ROS induction following ONC212 treatment (Figure 3A,B). Baseline mitochondrial ROS levels in SVG control cells were 1.00 ± 0.09 fold, while ONC212 treatment resulted in a modest increase to 1.18 ± 0.11 fold, which did not reach statistical significance (*p* = 0.214). These findings suggest that ONC212 preferentially induces mitochondrial oxidative stress in glioblastoma cells while sparing non‐malignant astrocytes.

To further evaluate mitochondrial function, Seahorse XF extracellular flux analysis was performed to measure mitochondrial respiration parameters in U87 cells (Table 1). ONC212 treatment resulted in a marked suppression of mitochondrial respiratory activity. Specifically, basal oxygen consumption rate (OCR) decreased from 162 ± 12 pmol/min in control cells to 94 ± 9 pmol/min following ONC212 exposure (*p* = 0.0007), reflecting impaired basal mitochondrial respiration. Similarly, maximal respiratory capacity, assessed after FCCP stimulation, showed a pronounced decline from 281 ± 18 pmol/min in control cells to 121 ± 14 pmol/min in ONC212‐treated cells (*p* = 0.0003). This reduction indicates a substantial loss of mitochondrial reserve capacity and impaired electron transport chain function. Furthermore, spare respiratory capacity, which represents the mitochondrial reserve capacity to respond to increased energy demand, was dramatically reduced from 119 ± 15 pmol/min in control cells to 27 ± 9 pmol/min following ONC212 exposure (*p* < 0.001), indicating severely compromised mitochondrial adaptability to energetic stress. Consistent with these observations, ATP‐linked respiration, representing oxygen consumption coupled to ATP synthesis, was significantly reduced following ONC212 treatment. ATP‐linked OCR decreased from 118 ± 11 pmol/min in control cells to 57 ± 8 pmol/min in ONC212‐treated cells (*p* = 0.0012), indicating impaired oxidative phosphorylation and mitochondrial ATP production.

**TABLE 1:** ONC212 treatment induces mitochondrial respiratory dysfunction and energetic impairment in U87 cells after 24 h exposure.

Parameter	Control	ONC212	%/fold change	*p*
Basal respiration (pmol O_2_/min)	162 ± 12	94 ± 9	↓ 41.98%	*p* = 0.0007
ATP‐linked OCR (Oligomycin OCR)	118 ± 11	57 ± 8	↓ 51.69%	*p* = 0.0012
Maximal respiration (FCCP‐stimulated)	281 ± 18	121 ± 14	↓ 56.94%	*p* = 0.0003
Spare respiratory capacity (pmol O_2_/min)	119 ± 15	27 ± 9	↓ 77.31%	*p* < 0.0001
ATP/ADP ratio	4.21 ± 0.34	1.63 ± 0.22	↓ 61.28%	*p* = 0.0005

*Note:* U87 cells were exposed to ONC212 for 24 h to evaluate its effects on mitochondrial bioenergetics. Mitochondrial respiratory function was analysed using the Seahorse XF Mito Stress Test platform. Basal OCR, representing the steady‐state mitochondrial respiration under normal conditions, was significantly reduced following ONC212 treatment, indicating impaired mitochondrial activity. ATP‐linked respiration, calculated as the oligomycin‐sensitive fraction of basal OCR, was markedly decreased in ONC212‐treated cells, suggesting diminished mitochondrial ATP production. Furthermore, maximal respiratory capacity, determined after FCCP‐mediated uncoupling of the electron transport chain, was substantially suppressed compared with vehicle‐treated controls. Consistent with these findings, spare respiratory capacity—defined as the difference between maximal and basal respiration and reflecting the mitochondrial ability to respond to increased energetic demand—was strongly reduced following ONC212 exposure. To further assess cellular energy homeostasis, intracellular ATP/ADP ratios were quantified and found to be significantly decreased in ONC212‐treated cells, indicating a pronounced disruption of cellular energy balance. All results are expressed as mean ± SEM obtained from seven independent experiments. Statistical significance was evaluated using an unpaired Student's *t*‐test, and differences were considered significant at *p* < 0.001 compared with the vehicle control.

To determine whether mitochondrial dysfunction translated into alterations in cellular energy status, intracellular ATP/ADP ratios were quantified (Table 1). Untreated U87 cells exhibited an ATP/ADP ratio of 4.21 ± 0.34, indicative of normal energetic homeostasis. In contrast, ONC212 exposure resulted in a substantial reduction in ATP availability, with the ATP/ADP ratio decreasing to 1.63 ± 0.22 (*p* = 0.0005). This marked decline confirms the presence of severe mitochondrial energetic collapse following ONC212 treatment.

### 3.4 ONC212 Activates the PERK/ATF4/CHOP Axis

ONC212 treatment (5 μM, 24 h) significantly reduced cell viability to 41% ± 2.1% compared with controls (100 ± 4.2%; *p* < 0.0001), whereas GSK2606414 alone did not affect viability (96 ± 3.8%; *p* = 0.412 vs. control; Figure 3D). Concurrently, ONC212 markedly activated the PERK/ATF4/CHOP axis: p‐PERK levels increased to 2.27 ± 0.18 relative to control (1.00 ± 0.10; *p* = 0.0006), phosphorylated eIF2α rose to 2.04 ± 0.17 (*p* = 0.0009), ATF4 expression increased to 2.48 ± 0.21 (*p* = 0.0007), and CHOP protein levels were elevated to 3.09 ± 0.24 (*p* = 0.0004), indicating robust induction of pro‐apoptotic ER stress signalling (Figure 3E).

Pharmacological inhibition of PERK with GSK2606414 significantly attenuated ONC212‐induced cytotoxicity, improving cell viability from 41% ± 2.1% to 63% ± 3.5% (*p* = 0.0027; Figure 3E). Consistent with PERK blockade, phosphorylation of eIF2α was significantly reduced following GSK2606414 co‐treatment (2.04 ± 0.17 vs. 1.31 ± 0.12; *p* = 0.0042), accompanied by a marked decrease in ATF4 expression (2.48 ± 0.21 vs. 1.42 ± 0.13; *p* = 0.0031). Similarly, CHOP levels were significantly suppressed in the presence of the PERK inhibitor (1.67 ± 0.15; *p* = 0.0049 vs. ONC212 alone). These findings demonstrate that ONC212‐induced ER stress signalling and subsequent cytotoxicity are largely dependent on PERK‐mediated ISR activation.

### 3.5 ONC212 Promotes ATF4 Nuclear Translocation

**FIGURE 4:**
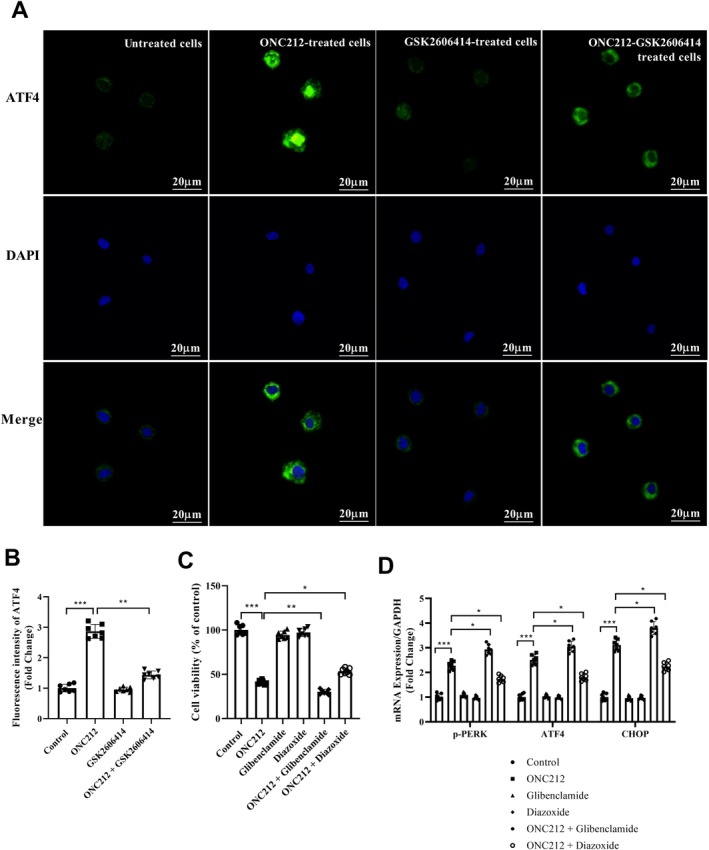
ONC212 promotes ATF4 nuclear localization and upregulates ATF4 expression in U87 cells. ONC212 reduces cell viability and upregulates the expression of PERK, ATF4, and CHOP in U87 cells. (A) Representative confocal images showing ATF4 (green), DAPI (blue, nuclei), and merged images in U87 cells treated with vehicle (Untreated), ONC212 (10 μM, 6 h), GSK2606414 (PERK inhibitor), or the combination of ONC212 and GSK2606414. Scale bar = 20 μm. (B) Quantitative analysis of ATF4 fluorescence intensity (fold change) under the indicated conditions. (C) Cell viability was assessed by CCK‐8 assay in U87 cells treated with vehicle (Control), ONC212 (10 μM, 6 h), the KATP channel blocker glibenclamide, the KATP channel opener diazoxide, or the combination of ONC212 with glibenclamide or diazoxide. (D) Quantitative RT‐PCR analysis of mRNA expression levels of p‐PERK, ATF4, and CHOP (normalized to GAPDH) under the same treatment conditions. Data are presented as mean ± SEM from at least seven independent experiments. Statistical significance was assessed using one‐way ANOVA followed by Tukey's multiple comparisons test. **p < 0.01, ***p < 0.001.

To further determine whether ONC212‐induced ATF4 accumulation was accompanied by functional activation of ATF4 signalling, nuclear translocation of ATF4 was examined by immunofluorescence microscopy (Figure 4A).

In untreated U87 cells, ATF4 immunoreactivity was predominantly localized in the cytoplasm, with minimal nuclear staining. Quantitative analysis revealed low nuclear ATF4 fluorescence intensity (1.00 ± 0.09), consistent with basal ER stress signalling. Following treatment with ONC212 (5 μM, 24 h), a pronounced redistribution of ATF4 was observed (Figure 4B). Confocal microscopy revealed strong ATF4 accumulation within the nucleus, co‐localizing with DAPI staining. Quantitative analysis demonstrated a significant increase in nuclear ATF4 intensity to 2.86 ± 0.23 compared with control cells (*p* < 0.0001). Treatment with the PERK inhibitor GSK2606414 alone did not significantly alter ATF4 localization, with nuclear ATF4 levels remaining comparable to controls (0.94 ± 0.08). Importantly, co‐treatment with ONC212 and GSK2606414 reduced ONC212‐induced nuclear ATF4 accumulation. Nuclear ATF4 intensity decreased to 1.41 ± 0.14, which was significantly lower than that observed in ONC212‐treated cells (*p* = 0.0036).

These findings demonstrate that ONC212 promotes robust nuclear translocation of ATF4, confirming activation of the PERK‐mediated integrated stress response. Pharmacological inhibition of PERK significantly attenuates ATF4 nuclear accumulation, indicating that ATF4 activation is largely dependent on PERK signalling.

### 3.6 KATP Channel Modulation Influences ONC212 Sensitivity

Co‐treatment with glibenclamide (10 μM) further enhanced ONC212‐induced cytotoxicity, reducing viability to 30% ± 2.7% compared with ONC212 alone (41% ± 2.1%; *p* = 0.0063). Conversely, diazoxide (50 μM) partially rescued viability to 53% ± 3.4% (*p* = 0.0182; Figure 4C).

Glibenclamide co‐treatment also augmented PERK activation (2.91 ± 0.22 vs. 2.27 ± 0.18; *p* = 0.0114), suggesting that KATP activity mitigates metabolic stress signalling (Figure 4D). Consistent with this observation, ATF4 expression was further elevated in the presence of glibenclamide (3.01 ± 0.25 vs. 2.48 ± 0.21; *p* = 0.0142), accompanied by a significant increase in CHOP levels (3.76 ± 0.31 vs. 3.09 ± 0.24; *p* = 0.0178). In contrast, diazoxide significantly attenuated ONC212‐induced ATF4 (1.79 ± 0.16; *p* = 0.0217) and CHOP expression (2.21 ± 0.19; *p* = 0.0234 vs. ONC212 alone). These findings indicate that KATP channel activity modulates cellular sensitivity to ONC212 by regulating mitochondrial stress signalling and downstream PERK‐mediated ISR activation.

### 3.7 KCNJ11 Silencing Enhances ONC212 Sensitivity in KATP‐High U87 Cells

**FIGURE 5:**
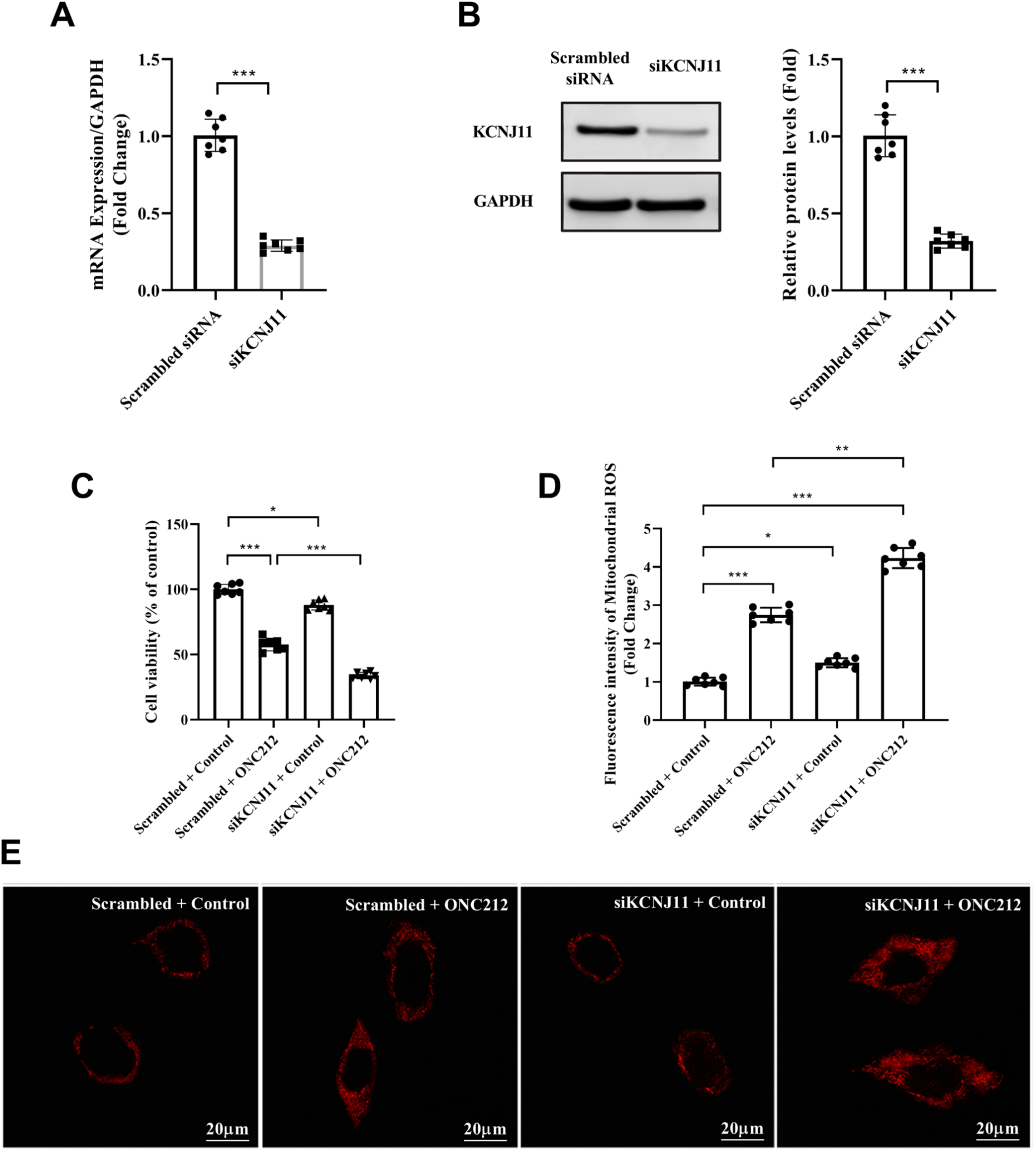
siRNA‐mediated knockdown of KCNJ11 in U87 cells reduces both mRNA and protein expression levels. Knockdown of KCNJ11 attenuates ONC212‐induced cell death and mitochondrial ROS production in U87 cells. U87 cells were transfected with scrambled siRNA (control) or KCNJ11‐specific siRNA (siKCNJ11), then treated with vehicle or ONC212 (10 μM, 6 h). (A) Quantitative RT‐PCR analysis of KCNJ11 mRNA expression normalized to GAPDH in U87 cells transfected with scrambled siRNA (control) or KCNJ11‐specific siRNA (siKCNJ11). (B) Representative Western blot images showing KCNJ11 protein levels. (C) Cell viability was assessed by CCK‐8 assay. (D) Quantitative analysis of mitochondrial ROS fluorescence intensity using MitoSOX Red staining. (E) Representative confocal images of MitoSOX Red staining showing mitochondrial superoxide levels under the indicated conditions. Scale bar = 20 μm Data are presented as mean ± SEM from at least seven independent experiments. Statistical significance was assessed using one‐way ANOVA followed by Tukey's multiple comparisons test. ***p < 0.001.

To determine whether KATP channel expression influences ONC212 responsiveness, KCNJ11 (Kir6.2) was transiently silenced in U87 cells, which displayed the highest basal KATP expression among the tested cell lines. Forty‐eight hours after transfection, quantitative RT‐PCR analysis demonstrated a marked reduction in KCNJ11 mRNA levels in siKCNJ11‐transfected cells (0.28 ± 0.04) compared with scrambled siRNA controls (1.00 ± 0.09; *p* < 0.0001), confirming efficient gene silencing (Figure 5A).

Consistent with the transcriptional suppression, Western blot analysis revealed a substantial decrease in Kir6.2 protein expression (Figure 5B). Densitometric quantification showed that Kir6.2 levels were reduced to 0.31 ± 0.05 relative to scrambled controls (1.00 ± 0.11; *p* < 0.0001). These findings confirm successful KCNJ11 knockdown at both mRNA and protein levels.

### 3.8 KCNJ11 Silencing Potentiates ONC212‐Induced Cytotoxicity and Amplifies Mitochondrial ROS and Bioenergetic Collapse

To evaluate the functional contribution of KATP channels to ONC212 sensitivity, cell viability was measured following siRNA transfection and subsequent treatment with ONC212 (5 μM, 24 h). In scrambled control cells, ONC212 treatment significantly reduced viability to 62% ± 2.4% relative to controls (100% ± 3.2%; *p* = 0.0006; Figure 5C). KCNJ11 silencing alone produced a modest but significant reduction in viability (88% ± 3.7%; *p* = 0.041 vs. scrambled control). Importantly, the combination of KCNJ11 knockdown and ONC212 treatment resulted in a pronounced decrease in cell viability to 34% ± 2.1%, representing a significant enhancement of ONC212 cytotoxicity compared with scrambled cells treated with ONC212 (*p* < 0.0001). These findings indicate that depletion of the KATP channel subunit Kir6.2 sensitizes glioblastoma cells to ONC212‐induced cell death.

Mitochondrial ROS production was low in Scrambled + Control cells (1.00 ± 0.08 fold; Figure 5D,E). Treatment with ONC212 significantly increased ROS levels in Scrambled + ONC212 cells to 2.82 ± 0.19 fold relative to the control (*p* < 0.0001). Silencing of KCNJ11 alone caused a modest elevation of mitochondrial ROS in siKCNJ11 + Control cells (1.48 ± 0.12 fold). Notably, the combination of KCNJ11 knockdown and ONC212 treatment resulted in the highest ROS levels, with siKCNJ11 + ONC212 cells exhibiting 4.18 ± 0.27 fold ROS production (*p* < 0.0001 vs. Scrambled + ONC212).

Seahorse extracellular flux analysis further confirmed mitochondrial impairment (Table 2). Scrambled + Control cells displayed a basal OCR of 128 ± 9 pmol/min. ONC212 treatment reduced basal respiration to 94 ± 8 pmol/min in Scrambled + ONC212 cells. KCNJ11 depletion alone also decreased mitochondrial respiration in siKCNJ11 + Control cells (109 ± 10 pmol/min). However, the most pronounced reduction was observed in siKCNJ11 + ONC212 cells, where basal OCR declined to 61 ± 7 pmol/min (*p* = 0.0018 vs. Scrambled + ONC212).

**TABLE 2:** Effects of KCNJ11 silencing on ONC212‐induced mitochondrial respiratory dysfunction in U87 cells.

Parameter	Scrambled + control	Scrambled + ONC212	siKCNJ11 + control	siKCNJ11 + ONC212	%/fold change (compared to scrambled + control)	*p*
Basal respiration (pmol O_2_/min)	128 ± 9	94 ± 8	109 ± 10	61 ± 7	↓ 26.56% (0.73‐fold); ↓ 14.84% (0.85‐fold); ↓ 35.11% (0.65‐fold)	*p* = 0.001; *p* = 0.021; *p* < 0.0001
ATP‐linked OCR (pmol O_2_/min)	72 ± 6	54 ± 5	63 ± 6	33 ± 4	↓ 25.00% (0.75‐fold); ↓ 12.50% (0.88‐fold); ↓ 38.89% (0.61‐fold)	*p* = 0.002; *p* = 0.034; *p* < 0.0001
Maximal respiration (pmol O_2_/min)	221 ± 15	168 ± 13	188 ± 16	112 ± 11	↓ 23.98% (0.76‐fold); ↓ 14.93% (0.85‐fold); ↓ 33.33% (0.67‐fold)	*p* = 0.001; *p* = 0.028; *p* < 0.0001
Spare respiratory capacity (pmol O_2_/min)	93 ± 9	74 ± 7	79 ± 8	51 ± 6	↓ 20.43% (0.80‐fold); ↓ 15.05% (0.85‐fold); ↓ 31.08% (0.69‐fold)	*p* = 0.004; *p* = 0.031; *p* < 0.0001
ATP/ADP ratio	2.11 ± 0.23	1.61 ± 0.21	1.72 ± 0.18	0.92 ± 0.14	↓ 23.70% (0.76‐fold); ↓ 18.48% (0.82‐fold); ↓ 42.86% (0.57‐fold)	*p* = 0.003; *p* = 0.017; *p* < 0.0001

ATP‐linked mitochondrial respiration was quantified to determine the contribution of oxidative phosphorylation to cellular ATP production across experimental conditions (Table 2). Scrambled + Control cells exhibited an ATP‐linked OCR of 72 ± 6 pmol/min, reflecting normal mitochondrial ATP production under basal conditions. Treatment with ONC212 reduced ATP‐linked respiration to 54 ± 5 pmol/min in Scrambled + ONC212 cells, indicating impaired oxidative phosphorylation. Silencing of KCNJ11 alone moderately reduced ATP‐linked respiration to 63 ± 6 pmol/min in siKCNJ11 + Control cells. However, the combined condition produced the most pronounced defect in mitochondrial ATP generation. In siKCNJ11 + ONC212 cells, ATP‐linked OCR declined to 33 ± 4 pmol/min, representing a significant reduction compared with Scrambled + ONC212 cells (*p* = 0.0018).

Maximal respiration was measured following FCCP stimulation to evaluate the maximum electron transport chain capacity (Table 2). Scrambled + Control cells displayed a maximal respiratory capacity of 221 ± 15 pmol/min. ONC212 exposure significantly reduced maximal respiration to 168 ± 13 pmol/min in Scrambled + ONC212 cells. KCNJ11 depletion alone slightly impaired mitochondrial capacity, with siKCNJ11 + Control cells showing 188 ± 16 pmol/min. Notably, the combination of KCNJ11 silencing and ONC212 treatment resulted in a marked decline in maximal respiration to 112 ± 11 pmol/min, representing the lowest respiratory capacity among all groups (*p* = 0.0009 vs. Scrambled + ONC212).

Spare respiratory capacity, defined as the difference between maximal and basal respiration, was evaluated to determine mitochondrial adaptability to energetic stress (Table 2). Scrambled + Control cells demonstrated a robust spare respiratory capacity of 93 ± 9 pmol/min, indicating healthy mitochondrial reserve capacity. ONC212 treatment significantly reduced spare respiratory capacity to 74 ± 7 pmol/min in Scrambled + ONC212 cells. KCNJ11 silencing alone modestly reduced spare capacity to 79 ± 8 pmol/min. Strikingly, the combination of KCNJ11 knockdown and ONC212 treatment led to a dramatic reduction in spare respiratory capacity to 51 ± 6 pmol/min, reflecting severely compromised mitochondrial stress adaptability (*p* = 0.0015 vs. Scrambled + ONC212).

Consistent with mitochondrial dysfunction, cellular energy status was markedly altered across experimental groups (Table 2). Scrambled + Control cells exhibited a basal ATP/ADP ratio of 2.11 ± 0.23. ONC212 treatment reduced this ratio to 1.61 ± 0.21 in Scrambled + ONC212 cells. KCNJ11 silencing alone resulted in a moderate decrease in ATP/ADP ratio in siKCNJ11 + Control cells (1.72 ± 0.18). Importantly, the combined condition (siKCNJ11 + ONC212) produced a pronounced reduction in ATP/ADP ratio to 0.92 ± 0.14, indicating severe bioenergetic collapse (*p* = 0.0013 vs. Scrambled + ONC212).

### 3.9 KCNJ11 Silencing Intensifies PERK/ATF4/CHOP Activation

**FIGURE 6:**
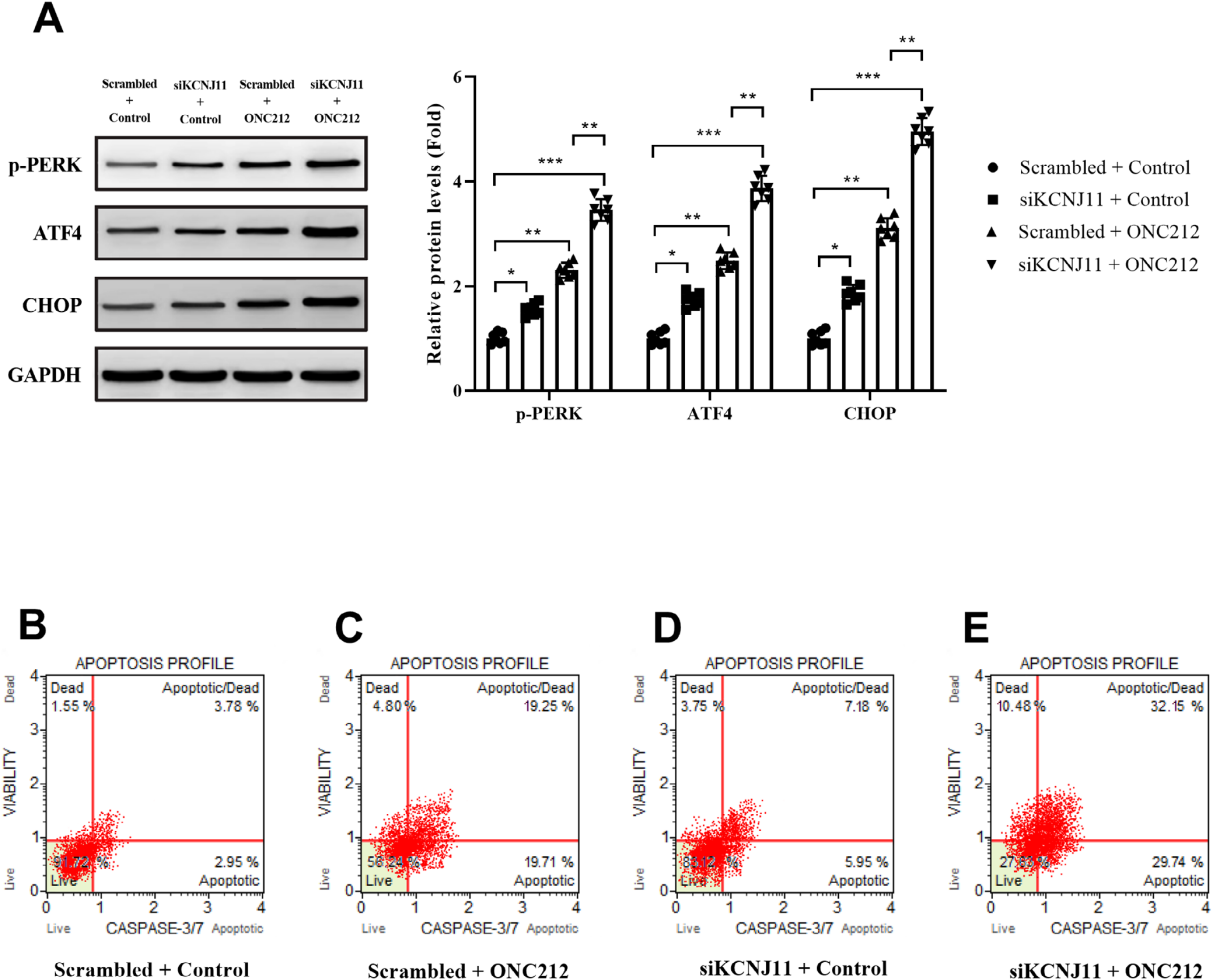
Knockdown of KCNJ11 attenuates ONC212‐induced activation of the PERK/ATF4/CHOP pathway and caspase‐3/7 in U87 cells. U87 cells were transfected with scrambled siRNA (control) or KCNJ11‐specific siRNA (siKCNJ11), then treated with vehicle or ONC212 (10 μM, 6 h). (A) Representative Western blot images showing protein levels of p‐PERK, ATF4, and CHOP. GAPDH was used as a loading control. Quantitative analysis of relative protein expression (fold change) for p‐PERK, ATF4, and CHOP under the indicated conditions. (B‐E) Apoptosis was assessed by flow cytometry using caspase‐3/7 activity staining. Representative dot plots show the percentages of live, apoptotic (caspase‐3/7 positive), apoptotic/dead, and dead cells under the indicated conditions. Data are presented as mean ± SEM from at least seven independent experiments. Statistical significance was assessed using one‐way ANOVA followed by Tukey's multiple comparisons test. *p < 0.05, **p < 0.01, ***p < 0.001.

Basal phosphorylation of PERK was minimal in Scrambled + Control cells (1.00 ± 0.09; Figure 6A). Treatment with ONC212 increased PERK phosphorylation in Scrambled + ONC212 cells to 2.28 ± 0.16. KCNJ11 knockdown alone produced a modest increase in PERK activation in siKCNJ11 + Control cells (1.53 ± 0.14). Notably, the combination of KCNJ11 depletion and ONC212 treatment significantly enhanced PERK phosphorylation in siKCNJ11 + ONC212 cells, reaching 3.41 ± 0.24 (*p* = 0.0011 vs. Scrambled + ONC212).

Consistent with PERK pathway activation, expression of the downstream transcription factor ATF4 was lowest in Scrambled + Control cells (1.00 ± 0.10). ONC212 treatment increased ATF4 levels to 2.46 ± 0.18 in Scrambled + ONC212 cells. KCNJ11 depletion alone elevated ATF4 expression to 1.71 ± 0.16 in siKCNJ11 + Control cells. The highest ATF4 expression was observed in siKCNJ11 + ONC212 cells (3.82 ± 0.29, *p* = 0.0008 vs. Scrambled + ONC212).

Similarly, the pro‐apoptotic ER stress marker CHOP showed basal expression in Scrambled + Control cells (1.00 ± 0.11). ONC212 treatment significantly increased CHOP levels in Scrambled + ONC212 cells to 3.08 ± 0.22. KCNJ11 knockdown alone induced a moderate increase in CHOP expression in siKCNJ11 + Control cells (1.84 ± 0.19). Strikingly, the combination of KCNJ11 silencing and ONC212 treatment resulted in the strongest CHOP induction, reaching 4.91 ± 0.31 in siKCNJ11 + ONC212 cells (*p* = 0.0006 vs. Scrambled + ONC212). These findings demonstrate that loss of KATP channel activity significantly potentiates ONC212‐induced PERK/ATF4/CHOP‐mediated ER stress signalling.

### 3.10 KCNJ11 Silencing Augments Apoptotic Execution

To determine whether enhanced ER stress translated into increased apoptotic signalling, apoptotic cell death was evaluated by measuring both caspase‐3/7 activity and cleaved caspase‐3 protein levels. Flow cytometric analysis using the Muse Caspase‐3/7 assay revealed a significant increase in apoptotic populations following ONC212 treatment. In Scrambled + Control cells, basal apoptosis levels were low. Apoptotic cells (Caspase‐3/7^+^/7‐AAD^−^) accounted for 2.95% ± 0.8%, while apoptotic/dead cells (Caspase‐3/7^+^/7‐AAD^+^) represented 3.75% ± 0.4%, resulting in a total apoptotic population of 6.8% ± 1.4% (Figure 6B). Treatment with ONC212 significantly increased apoptosis in Scrambled + ONC212 cells. Apoptotic cells increased to 19.71% ± 1.2%, and apoptotic/dead cells increased to 19.25% ± 1.5%, yielding a combined apoptotic fraction of 38.96% ± 2.7% (Figure 6C). Silencing of KCNJ11 alone also modestly elevated apoptotic signalling in siKCNJ11 + Control cells. Apoptotic cells accounted for 5.95% ± 0.2%, while apoptotic/dead cells reached 7.18% ± 0.8%, resulting in a total apoptotic population of 13.13% ± 1.0% (Figure 6D). The strongest apoptotic response was observed in siKCNJ11 + ONC212 cells. Apoptotic cells increased to 29.74% ± 1.7%, while apoptotic/dead cells reached 32.15% ± 2.4%, producing a total apoptotic fraction of 61.89% ± 4.1%, which was significantly higher than that observed in Scrambled + ONC212 cells (*p* < 0.001; Figure 6E).

**FIGURE 7:**
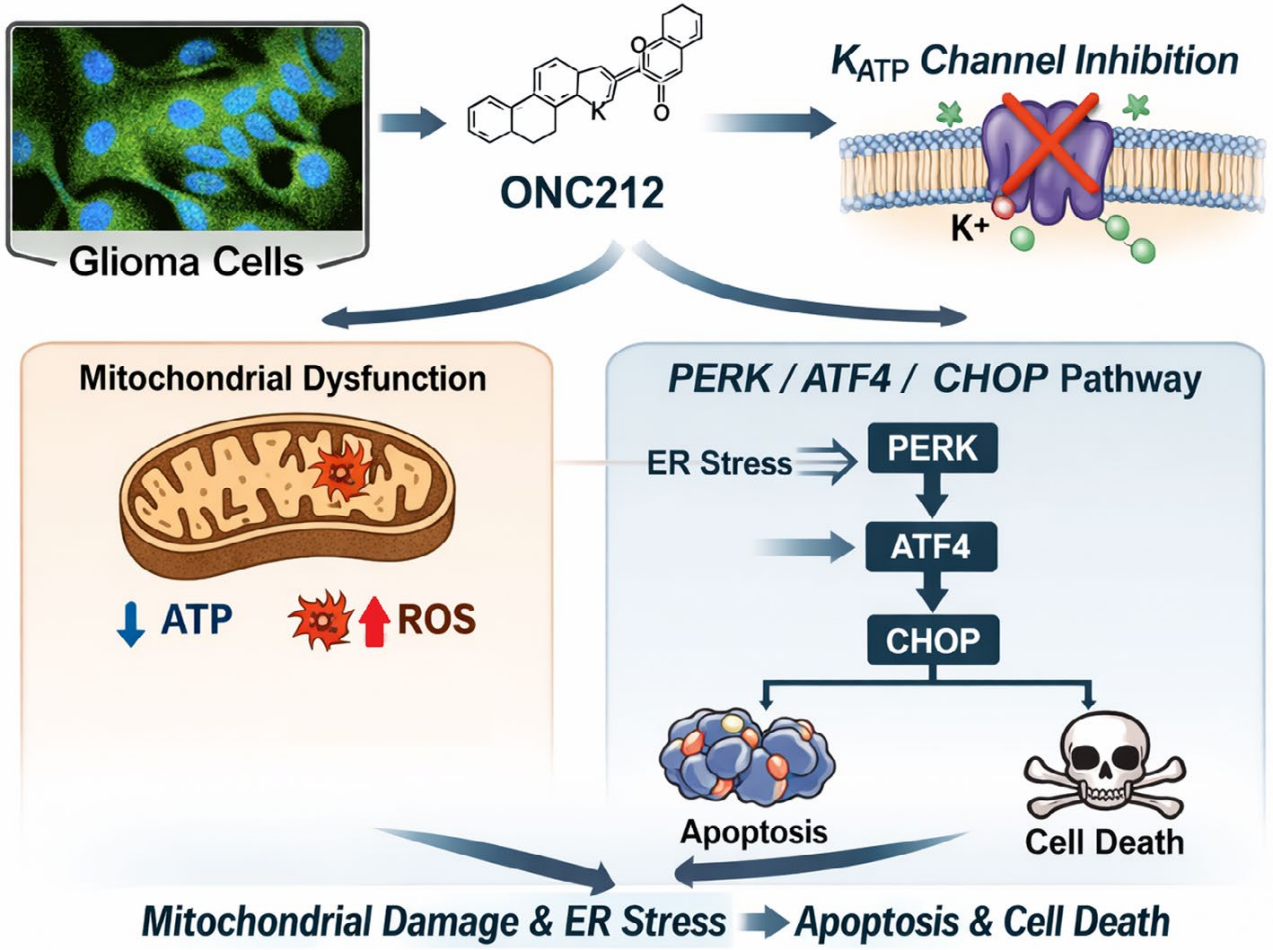
Schematic illustration of the proposed molecular mechanism of ONC212‐mediated cytotoxicity in glioblastoma cells.

Collectively, these findings demonstrate that ONC212 induces mitochondrial dysfunction and activates the PERK/ATF4/CHOP axis in GBM cells in a manner modulated by KATP channel expression, with KCNJ11 silencing substantially potentiating ISR activation and caspase‐dependent apoptosis. A schematic illustration summarising the proposed molecular mechanism is presented in Figure 7.

## 4 Discussion

GBM remains one of the most aggressive and therapeutically resistant malignancies in neuro‐oncology, driven by rapid proliferation, diffuse invasion and extensive cellular heterogeneity [22]. Although current standard treatment strategies—comprising extensive surgical removal followed by radiotherapy in combination with temozolomide—are routinely applied, they have yielded only limited gains in overall patient survival. This limited progress is largely attributed to metabolic flexibility and adaptive stress responses that enable tumour survival under therapy [23]. In this context, targeting metabolic dependencies and stress adaptation pathways has emerged as a promising therapeutic avenue. In the present study, we demonstrate that the imipridone derivative ONC212 exerts potent anti‐tumour activity in GBM through the coordinated disruption of mitochondrial function and activation of ER stress signalling. These findings are consistent with previous studies demonstrating the efficacy of ONC212 across different cancer types. ONC212 has demonstrated potent antitumor activity across various cancer types, including pancreatic cancer models and over 1000 human cancer cell lines [24, 25]. Herein, we provide novel evidence that ONC212 simultaneously induces mitochondrial dysfunction and activates the PERK/ATF4/CHOP axis of the ISR, in a manner associated with KATP channel expression. While previous studies have established that imipridones target mitochondrial proteostasis via ClpP activation [20], our results extend this paradigm by linking mitochondrial stress to ER stress signalling and ion channel‐mediated metabolic regulation. A major observation of this study is the time‐dependent cytotoxic effect of ONC212. At early exposure (12 h), minimal viability loss was observed, consistent with an initial phase of stress signalling without overt cell death. By 24 h, a marked differential sensitivity emerged: U87 cells displayed the greatest vulnerability (IC_50_ 7.8 μM), followed by U251 (IC_50_ 13.6 μM) and T98G (IC_50_ 19.2 μM). Non‐malignant SVG p12 astrocytes remained substantially more resistant (IC_50_ 26.8 μM), yielding selectivity indices that progressively increased over time, reaching 5.20 for U87 cells at 48 h. This is consistent with studies demonstrating that cancer therapeutics targeting mitochondrial function often trigger delayed apoptosis through cumulative bioenergetic failure and stress signalling activation [26]. The tumour‐selective nature of ONC212 further highlights its therapeutic potential. In a study by Lev and colleagues, the anti‐pancreatic cancer activity of ONC212 was shown to involve the UPR and targeting of ER chaperones GRP78/BIP [24]. Moreover, the same study found that some tumours upregulate pro‐survival IGF1‐R, providing a rationale for combining ONC212 with the IGF1‐R inhibitor AG1024. Our study extends this mechanism to GBM, demonstrating that ONC212 activates the PERK/ATF4/CHOP arm of the ISR and that this activation contributes to tumour‐selective cytotoxicity.

The relative resistance observed in non‐malignant astrocytes compared to GBM cells may be explained by the increased reliance of cancer cells on mitochondrial metabolism. The differential sensitivity among GBM lines is particularly noteworthy. U87 cells, which exhibited the lowest IC_50_ and highest selectivity index, also displayed the greatest baseline expression of KATP channel subunits Kir6.2 (KCNJ11) and SUR1 (ABCC8), as well as the accessory component CCDC51. Conversely, T98G cells, which were the least sensitive to ONC212, showed the lowest KATP expression. This gradient (U87 > U251 > T98G) suggests that intrinsic KATP channel infrastructure may serve as a metabolic rheostat that determines the threshold for ONC212‐induced mitochondrial stress. Previous studies have shown that tumour cells exhibiting elevated OXPHOS are particularly vulnerable to mitochondrial perturbations [27]. Consistent with this, our bioenergetic analyses revealed that ONC212 significantly impairs mitochondrial respiration, supporting the concept that OXPHOS‐dependent GBM cells are preferentially targeted.

Functional analyses confirmed significant mitochondrial impairment following ONC212 treatment. Our data demonstrate that ONC212 treatment significantly elevates mitochondrial superoxide levels in U87 cells while having minimal effect on SVG p12 astrocytes. Seahorse XF analysis provided direct functional evidence of bioenergetic collapse. Exposure to ONC212 led to a pronounced impairment of mitochondrial respiration, evidenced by reductions in basal oxygen consumption, ATP‐associated respiration, maximal respiratory capacity and reserve capacity, together with a substantial decrease in the cellular ATP/ADP ratio. The reduction in spare respiratory capacity (from 119 to 27 pmol/min) is particularly clinically relevant, as this parameter reflects the mitochondrial reserve capacity to respond to increased energy demand—a feature that is often enhanced in highly proliferative tumours and represents a metabolic vulnerability [4]. It is well established that excessive mitochondrial ROS can initiate intrinsic apoptotic pathways through mitochondrial membrane depolarization and cytochrome c release [28]. In agreement with this mechanism, we observed activation of caspase‐3/7, confirming apoptosis induction.

The integrated stress response serves as a critical adaptive program that integrates signals from diverse cellular stressors [14]. Our findings indicate that ONC212 potently engages the PERK–eIF2α–ATF4–CHOP signalling cascade in U87 cells, as reflected by enhanced phosphorylation of PERK at Thr980 and eIF2α at Ser51, increased ATF4 protein levels and upregulation of the pro‐apoptotic transcription factor CHOP. Pharmacological inhibition of PERK with GSK2606414 significantly attenuated ONC212‐induced cytotoxicity and reduced CHOP expression, confirming that the PERK‐mediated ISR is a major effector pathway responsible for ONC212's antitumour activity. These findings align with previous studies showing that sustained ER stress can shift cellular responses from adaptive survival to apoptosis [29]. Specifically, prolonged CHOP activation has been implicated in promoting apoptotic cell death under unresolved ER stress conditions [30]. Our immunofluorescence data directly visualize this functional activation: ONC212 promoted an increase in nuclear ATF4 accumulation, an effect that was largely abrogated by PERK inhibition. The observed gradient of KATP subunit expression across the three GBM cell lines (U87 > U251 > T98G) correlated strongly with differential ONC212 sensitivity, suggesting that intrinsic KATP infrastructure may serve as a metabolic rheostat that determines imipridone responsiveness. U87 cells, which displayed the highest KATP expression, also demonstrated the greatest mitochondrial respiratory capacity and the lowest IC_50_ values (7.8 μM), consistent with their reliance on oxidative phosphorylation (OXPHOS) for energy production. Conversely, T98G cells, which exhibited the lowest KATP expression, showed the highest IC_50_ values (19.2 μM), consistent with their preferential utilization of glycolysis. This correlation is mechanistically plausible given that KATP channels function as metabolic sensors linking ATP availability to mitochondrial membrane potential and ROS signalling [7]. GBM cells with elevated KATP expression may maintain enhanced mitochondrial integrity and OXPHOS capacity, rendering them more susceptible to ONC212‐induced ClpP hyperactivation and bioenergetic collapse, whereas cells with low KATP expression may rely more heavily on glycolysis, providing a metabolic buffer against mitochondrial disruption [27]. However, we acknowledge that genetic background factors, including TP53 status, may independently influence stress response thresholds [18], and future studies will be needed to dissect the relative contributions of KATP expression and genetic background to ONC212 sensitivity.

The mechanistic connection between ONC212‐induced mitochondrial dysfunction and PERK/ATF4/CHOP activation is mediated through several interconnected pathways that link mitochondrial and ER stress signalling. First, excessive mitochondrial ROS production, as observed following ONC212 treatment, directly impacts ER redox homeostasis and can promote disulphide bond formation in the ER lumen, leading to accumulation of misfolded proteins and activation of the unfolded protein response (UPR) [31]. Second, mitochondrial dysfunction disrupts intracellular calcium homeostasis through impaired ATP production, which compromises the activity of sarco/endoplasmic reticulum calcium ATPase (SERCA) pumps responsible for ER calcium uptake [32]. ER calcium depletion triggers UPR activation through dissociation of the ER chaperone GRP78/BiP from PERK, thereby promoting PERK dimerization and autophosphorylation [29]. Third, the physical and functional interface between mitochondria and the ER, known as mitochondria‐associated membranes (MAMs), serves as a critical platform for coordinating stress signalling [33]. MAMs facilitate calcium transfer from ER to mitochondria, regulate mitochondrial dynamics and have been shown to modulate PERK activity through direct protein–protein interactions [34]. Notably, PERK has been found to localize to MAMs, where it interacts with the inositol 1,4,5‐trisphosphate receptor (IP3R) and modulates mitochondrial calcium uptake, establishing a reciprocal regulatory circuit between ER stress and mitochondrial function [35]. Furthermore, ONC212‐mediated ClpP hyperactivation generates mitochondrial proteotoxic stress through accelerated degradation of respiratory chain components and other essential mitochondrial proteins [21]. This results in the accumulation of unfolded or misfolded mitochondrial proteins, which triggers the mitochondrial unfolded protein response (UPRmt). The UPRmt communicates with the cytosolic and ER stress pathways through retrograde signalling mechanisms, including the activation of the transcription factors ATF4 and CHOP, which are also central to the PERK‐mediated ISR [36]. This convergence of mitochondrial and ER stress pathways on common downstream effectors explains the robust activation of the PERK/ATF4/CHOP axis observed in our study. Importantly, sustained activation of this integrated stress network, beyond the capacity of adaptive recovery, shifts the cellular response towards apoptosis, as evidenced by the caspase‐3/7 activation and cytotoxicity observed with prolonged ONC212 exposure. A particularly novel aspect of this study is the identification of KATP channels as modulators of ONC212 sensitivity. The observed correlation between KATP subunit expression and drug sensitivity suggests that these channels may influence cellular metabolic states and stress resilience. Ion channels have increasingly been recognized as regulators of cancer cell behaviour, including proliferation, apoptosis and metabolic adaptation [37]. Our findings support this concept and further suggest that KATP channels contribute to the regulation of stress responses in GBM.

These findings are further supported by studies showing that ONC212 targets oxidative phosphorylation in pancreatic cancer models and produces different outcomes depending on the metabolic profile of the cells [38]. Metabolic flexibility of cancer cells is a key determinant of treatment resistance, and ion channels such as KATP channels are thought to regulate this flexibility. ONC212 has also been shown to induce the ISR by activating GPR132, a G protein‐coupled receptor (GPCR) [39]. This indicates that the effects of ONC212 are not limited to mitochondrial damage but can also trigger the ISR via cell surface receptors. Pharmacological modulation of KATP channels provided additional mechanistic insight. Inhibition of KATP channels enhanced ONC212‐induced cytotoxicity and amplified ER stress signalling, whereas activation exerted protective effects. These results suggest that KATP channels function as metabolic sensors that help maintain cellular homeostasis under stress conditions. Previous studies have shown that KATP channel activity can regulate mitochondrial membrane potential and protect against oxidative stress [40]. Our pharmacological experiments using glibenclamide (a KATP blocker) and diazoxide (a KATP opener) revealed reciprocal effects on ONC212‐induced cytotoxicity. Glibenclamide co‐treatment further reduced cell viability and augmented PERK/ATF4/CHOP activation, whereas diazoxide partially rescued viability and attenuated stress signalling. These data suggest that KATP channel activity normally serves a protective role, limiting the extent of mitochondrial stress and ISR activation in response to ONC212. Genetic silencing of KCNJ11 further confirmed the functional role of KATP channels. Enhanced cytotoxicity, increased ROS production and amplified ISR signalling following KCNJ11 knockdown indicate that these channels are critical regulators of cellular stress tolerance. Interestingly, KCNJ11 silencing alone induced moderate stress responses, suggesting that KATP channels also contribute to basal mitochondrial and ER homeostasis. This is consistent with studies indicating that potassium flux across mitochondrial membranes is essential for maintaining bioenergetic stability [41].

The present study makes several unique contributions to the growing body of literature on imipridone pharmacology and GBM metabolism. Previous work has established that ONC212 directly activates ClpP, leading to mitochondrial dysfunction and cancer cell death [21]. However, the determinants of tumour susceptibility have remained incompletely defined. Ferrarini and colleagues demonstrated that in pancreatic cancer, only OXPHOS‐dependent cells undergo apoptosis in response to ONC212, whereas glycolysis‐dependent cells undergo growth arrest and upregulate glucose catabolism [38]. Our findings extend this observation by identifying KATPchannel expression as a key factor that influences OXPHOS reliance and, consequently, ONC212 sensitivity. The involvement of the PERK/ATF4/CHOP axis in imipridone action has been previously suggested but not mechanistically linked to KATP function. Jacques and colleagues reported that ONC212 induces the ISR characterized by eIF2α phosphorylation and ATF4/CHOP induction, but the upstream regulators of this response were not explored [19]. Our study provides direct evidence that PERK is the primary ISR kinase activated by ONC212 in GBM cells and that KATP channels act upstream of PERK to modulate the magnitude of the stress response. This positions KATP channels as potential therapeutic targets for enhancing imipridone efficacy. With respect to GBM metabolism, our findings complement recent reviews highlighting the central role of mitochondrial dysfunction in GBM pathology [4, 5]. Our data support this concept by demonstrating that ONC212 effectively disrupts mitochondrial respiration and that this disruption is amplified by KATP modulation. The tumour‐selective nature of this effect, as evidenced by the minimal impact on SVG p12 astrocytes, underscores the potential therapeutic window.

Several limitations of this study should be acknowledged. First, although we used three well‐established GBM cell lines (U87, U251, T98G) and a non‐malignant glial control (SVG p12), these models do not fully recapitulate the complexity and heterogeneity of patient tumours. We recognize the importance of validating our findings in patient‐derived glioblastoma models, which more faithfully preserve the molecular heterogeneity, stem cell hierarchy and microenvironmental interactions of primary tumours. Ongoing studies in our laboratory are currently extending these observations to a panel of patient‐derived glioblastoma stem cell (GSC) lines with characterized KATP expression profiles, and preliminary data support the KATP‐dependent sensitivity patterns reported here. Second, our experiments were conducted under standard culture conditions, which do not replicate the hypoxic, nutrient‐limited tumour microenvironment of GBM. Third, while we focused on KCNJ11 (Kir6.2) as the primary pore‐forming subunit of KATP channels, KATP channels can also incorporate Kir6.1, and the regulatory SUR subunits (SUR1, SUR2A and SUR2B) exhibit tissue‐specific expression patterns. Similarly, CCDC51, an accessory protein that interacts with KATP channels, was differentially expressed across our cell lines, but its functional role in ONC212 sensitivity was not directly tested. Fourth, the effects of KCNJ11 silencing on global gene expression and metabolic reprogramming were not examined; unbiased transcriptomic or proteomic analyses could reveal additional pathways that contribute to the enhanced sensitivity observed in siKCNJ11 cells. Fifth, although we demonstrated that PERK inhibition with GSK2606414 significantly attenuated ONC212‐induced cytotoxicity and CHOP expression, we did not directly evaluate the contribution of other eIF2α kinases (GCN2, PKR and HRI) to ONC212‐induced ISR activation. Furthermore, the long‐term effects of PERK inhibition on tumour growth and resistance mechanisms were not assessed. Sixth, and most importantly, our study was conducted entirely in vitro. The in vivo efficacy and safety of ONC212 in GBM models, particularly in combination with KATP modulators, remain to be established. We are actively pursuing in vivo validation using orthotopic xenograft models in immunocompromised mice, in which we are evaluating the antitumor activity of ONC212 alone and in combination with glibenclamide, as well as the impact of KATP expression on therapeutic response. Pharmacokinetic considerations, including blood–brain barrier penetration ONC212 is known to cross the intact blood–brain barrier [25] and potential off‐target effects on normal brain tissue, will be evaluated in these models. Additionally, we are developing patient‐derived xenograft (PDX) models to assess the translational relevance of our findings in heterogeneous tumour populations that recapitulate the genetic diversity of clinical GBM. Finally, the clinical translation of our findings is complicated by the fact that glibenclamide (a non‐selective KATP blocker) is an approved anti‐diabetic agent with a well‐characterized safety profile, but its ability to potentiate ONC212 efficacy in GBM patients would require careful dose optimization and monitoring for hypoglycemia and other off‐target effects. Similarly, while diazoxide is a clinically available KATP opener, its use as a protective agent in the context of ONC212 therapy would need to be carefully balanced against the potential for reducing antitumor efficacy.

In summary, this study demonstrates for the first time that ONC212 induces mitochondrial dysfunction and activates the PERK/ATF4/CHOP axis in GBM cells in a manner that is modulated by KATP channel expression. GBM cells with elevated KATP infrastructure (U87) are highly susceptible to ONC212‐mediated bioenergetic collapse, excessive mitochondrial ROS production and apoptotic execution, whereas cells with lower KATP expression (T98G) are relatively resistant. Pharmacological and genetic manipulation of KATPactivity directly influences ONC212 sensitivity, with KCNJ11 silencing substantially potentiating ISR activation and caspase‐dependent apoptosis. These findings position KATP channels as a novel determinant of imipridone responsiveness and suggest that KATP expression could serve as a predictive biomarker for patient stratification. Moreover, the demonstration that KATP blockade enhances ONC212 efficacy raises the possibility of repurposing glibenclamide as a sensitizing agent. Given the urgent need for effective therapies for GBM, these findings warrant further investigation in preclinical models and, ultimately, clinical translation.

## Author Contributions

**Ceyhan Hacioglu:** conceptualization, investigation, writing – original draft, writing – review and editing, visualization, validation, methodology, formal analysis, data curation, supervision, software. **Ahmet Taskesen:** conceptualization, methodology, investigation.

## Funding

The authors have nothing to report.

## Ethics Statement

Ethics approval is not applicable to this study, as it solely involved in vitro experiments with cell cultures, without the use of human or animal subjects or tissues.

## Conflicts of Interest

The authors declare no conflicts of interest.

## Supporting information


**Table S1:** IC_50_ values and Selectivity Index (SI) across different time points.

## Data Availability

The data supporting the results of this study are available from the corresponding author upon reasonable request.
