# Supplementary material for: KATP Channel Expression Determines ONC212 Sensitivity via Mitochondrial Dysfunction and PERK/ATF4/CHOP Activation in Glioblastoma
Source: J Cell Mol Med. 2026 Jul 25;30(14):e71301. doi: 10.1111/jcmm.71301 (PMC13401692; doi:10.1111/jcmm.71301)
Supplement: Supplementary file 1 — Table S1: IC50 values and Selectivity Index (SI) across different time points. [file JCMM-30-e71301-s001.docx]

**Table S1.** IC₅₀ values and Selectivity Index (SI) across different time points

| **Time (h)** | **Cell Line** | **IC₅₀ (µM)** | **SI (SVG / Cancer)** |
| --- | --- | --- | --- |
| 12 | U87 | 28.4 ± 2.1 | >1.76 |
|  | U251 | 34.6 ± 2.8 | >1.44 |
|  | T98G | 41.2 ± 3.4 | >1.21 |
| 24 | U87 | 7.8 ± 0.5 | 3.44 |
|  | U251 | 13.6 ± 0.9 | 1.97 |
|  | T98G | 19.2 ± 1.2 | 1.40 |
| 48 | U87 | 4.5 ± 0.4 | 5.20 |
|  | U251 | 9.1 ± 0.7 | 2.57 |
|  | T98G | 15.2 ± 1.1 | 1.54 |
| — | SVG | >50 (12 h) | — |
|  |  | 26.8 (24 h) | — |
|  |  | 23.4 (48 h) | — |
